# ArchCaries-YOLO26: a dentition-aware framework for automated detection of primary and permanent tooth caries in intraoral photographs

**DOI:** 10.3389/fmed.2026.1955589

**Published:** 2026-10-13

**Authors:** Zhenwei Guo, Shiqian Wu, Peipei Zhang, Di Xue, Zhuo Chen

**Affiliations:** Department of Stomatology, Zhengzhou Central Hospital Affiliated to Zhengzhou University, Zhengzhou, China

**Keywords:** deep learning, dental caries, dental-arch modeling, intraoral photographs, object detection

## Abstract

To address the challenges of small lesion scales, irregular boundaries, substantial background interference, and similar local appearances between caries in primary and permanent teeth in color intraoral images, this paper proposes ArchCaries-YOLO26, a YOLO26-based object detection model for dental caries. Specifically, this study addresses the problem of accurately detecting and localizing carious lesions in intraoral photographs while distinguishing whether the detected lesions occur in primary or permanent teeth. The model introduces the Adaptive Dental-Arch Trajectory State-Space Module (ADT-SSM), which models the arrangement, scale, and morphological relationships of adjacent teeth through bidirectional state propagation along predicted dental-arch trajectories. Meanwhile, the Class-Conditioned Inner–Outer Boundary Integral Calibration module (CIBC) is designed to adaptively refine the bounding-box boundaries of caries in primary and permanent teeth by jointly analyzing the features of the inner candidate region, boundary ring, and outer dental tissue. On the public dataset, the proposed model achieves mAP@50, mAP@50-95, Precision, and Recall values of 0.951±0.006, 0.799±0.007, 0.918±0.005, and 0.872±0.006, respectively. On the clinical dataset, the corresponding metrics reach 0.920±0.006, 0.776±0.007, 0.779±0.009, and 0.905±0.009, respectively. The ablation results demonstrate that ADT-SSM and CIBC play complementary roles in dentition-context modeling and bounding-box localization, and their joint use achieves a more balanced performance in classification and localization. The proposed method provides a feasible technical approach for the automated screening of caries in primary and permanent teeth using conventional color intraoral images.

## 1 Introduction

Dental caries is one of the most common oral diseases, and its development and progression may lead to the destruction of dental hard tissues, pain, impaired masticatory function, and subsequent pulpal and periapical lesions (1, 2). Timely identification and localization of carious teeth are important for early intervention and the formulation of targeted treatment plans. Conventional caries diagnosis primarily relies on clinical visual examination, probing, and dental imaging, which impose certain requirements on professional expertise, examination equipment, and operating environments (3, 4). With the development of mobile imaging devices and computer vision technologies, color intraoral images have gradually become an important data source for auxiliary oral health screening because they can be conveniently acquired using relatively low-cost devices and directly present the appearance of tooth surfaces. Deep learning-based object detection methods can simultaneously provide caries categories and spatial locations, thereby offering interpretable visual information for primary-level screening, oral health education, and clinical examination assistance. Therefore, they possess considerable research significance and application potential.

However, dental caries detection in color intraoral images still faces multiple challenges. Carious lesions are generally characterized by small sizes, irregular morphologies, indistinct boundaries, and insufficient local contrast (5). Their appearances can be easily confused with dental pits and fissures, interdental spaces, tooth-surface pigmentation, illumination-induced shadows, and saliva reflections. Variations in imaging viewpoints, lighting conditions, and occlusion by oral soft tissues can also cause substantial shifts in image distributions, increasing the difficulty of lesion feature extraction and boundary localization (6). Meanwhile, primary and permanent teeth may coexist during the mixed-dentition stage. Although carious regions in these two dentition types may exhibit highly similar colors and textures, they differ in crown size, inter-tooth distance, adjacency relationships, and eruption status. Existing detection networks generally aggregate features over regular two-dimensional grids and therefore cannot adequately exploit the continuous structural information of teeth arranged along curved dental arches (7). In addition, conventional detection heads primarily regress rectangular bounding boxes directly from local features, without explicitly modeling the differences among lesions inside candidate boxes, transitional regions around their boundaries, and normal dental tissues outside the boxes.

To address these issues, this paper develops ArchCaries-YOLO26 for dental caries detection in color intraoral images and improves feature representation and localization from the perspectives of dentition-context modeling and candidate-box boundary calibration. The model introduces the Adaptive Dental-Arch Trajectory State-Space Module (ADT-SSM), which predicts local dental-arch directions, displacements, and curvatures to construct learnable curved sampling paths on feature maps. Bidirectional state propagation is then performed along these dental-arch trajectories to aggregate information regarding the arrangement, size, and morphology of potentially adjacent teeth. The model also incorporates the Class-Conditioned Inner–Outer Boundary Integral Calibration module (CIBC). Based on each initial candidate box, CIBC constructs an inner region, a boundary-ring region, and an outer region. It then combines the soft class probabilities of primary-tooth and permanent-tooth caries to adaptively refine the left, top, right, and bottom boundaries of the detection box. The two modules operate on structural semantic modeling and spatial boundary optimization, respectively, enabling local caries information to be jointly represented with dentition relationships and surrounding dental context.

The main contributions of this paper are summarized as follows:

- We propose ADT-SSM, which transforms feature propagation over regular grids into bidirectional state propagation along learnable dental-arch trajectories. Without requiring additional tooth segmentation masks or tooth-position annotations, ADT-SSM models the structural continuity of the dentition and provides contextual information for distinguishing primary-tooth caries from permanent-tooth caries.
- We propose CIBC, which jointly analyzes lesion evidence inside candidate boxes, transitional information around their boundaries, and normal dental-tissue features outside the boxes. By using class probabilities to control four-directional boundary-offset calibration, CIBC improves the model’s ability to represent the bounding-box boundaries of small, irregular, and low-contrast carious regions.
- We develop an end-to-end dental caries detection framework that integrates dental-arch trajectory modeling with inner–outer boundary calibration. This framework establishes a complementary relationship between dentition-level contextual representation and candidate-box-level localization optimization, providing a unified approach for detecting caries in primary and permanent teeth from color intraoral images.

## 2 Related work

### 2.1 Deep learning-based dental caries detection

In recent years, deep learning techniques have been increasingly applied to the automatic detection of dental caries in dental images, including periapical radiographs, bitewing radiographs, panoramic radiographs, near-infrared transillumination images, and localized dental X-ray images. Lee et al. employed a convolutional neural network to detect and diagnose dental caries in periapical radiographs, demonstrating the potential of deep features for identifying dental lesions (8). Schwendicke et al. further applied deep learning to near-infrared light transillumination images and explored an automated caries lesion detection method under non-ionizing imaging conditions (9). For bitewing radiographs, which are commonly used in clinical practice, Cantu et al. investigated the deep learning-based detection of caries lesions with different radiographic extents, showing that the model could distinguish lesions with varying depths of progression (10). Lee et al. used deep learning for early dental caries detection and evaluated the influence of artificial intelligence-assisted results on clinical diagnostic performance (11). More recently, Balachandran and Marappan proposed EnamelNet-TRiX, a lesion-aware dual-Transformer architecture with cross-attention for the diagnosis of early and advanced enamel caries (12). Marappan et al. also investigated AI-driven approaches for enhancing the early detection of dental caries, further demonstrating the growing role of artificial intelligence in caries screening and diagnosis (13). In other dental X-ray imaging scenarios, Vinayahalingam et al. applied deep learning to classify third-molar caries in panoramic radiographs (14). Bayraktar and Ayan employed a deep convolutional neural network to localize proximal caries lesions in digital bitewing radiographs (15). Chen et al. adopted an object detection framework to identify proximal caries lesions of different severity levels (16), whereas Ying et al. integrated multi-scale features with a deep network architecture to segment carious regions in dental X-ray images (17). These studies demonstrate that deep learning can learn discriminative lesion representations from complex dental images and has promoted the development of dental caries analysis from image-level classification to lesion localization and pixel-level segmentation.

With the development of smartphones and portable imaging devices, dental caries detection based on color intraoral photographs has gradually become an important research direction for low-cost screening. Zhang et al. developed a deep learning-based screening model for oral photographs and used an object detection method to identify and localize carious regions, providing a feasible solution for automated screening with non-professional imaging devices (18). Park et al. first segmented tooth surfaces in intraoral photographs and subsequently performed caries classification and localization, thereby reducing interference from the gingiva, tongue, and other oral background tissues (19). Adnan et al. further combined YOLO and Transformer-based detection frameworks to develop an artificial intelligence application for caries index detection in intraoral photographs, demonstrating the potential of deep learning models for mobile oral health screening (20). Meanwhile, research on complex caries types and advanced detection architectures has continued to progress. Chaves et al. used deep learning to detect secondary caries around restorations in bitewing radiographs, extending the applicability of these models to more challenging clinical scenarios (21). Bayati et al. employed YOLOv8 to identify proximal caries lesions in bitewing radiographs, further validating the effectiveness of advanced single-stage detectors for dental lesion localization (22). Although existing methods have achieved promising detection performance, color intraoral images remain affected by uneven illumination, saliva-induced reflections, soft-tissue interference, densely arranged teeth, variations in lesion scale, and indistinct caries boundaries. Therefore, enhancing fine-grained lesion features, sufficiently integrating multi-scale information, and suppressing background interference in complex oral environments remain critical challenges for caries object detection based on intraoral photographs.

### 2.2 Object detection methods for intraoral dental images

With the development of intraoral imaging devices and deep learning techniques, object detection methods have been increasingly applied to the automatic localization of teeth, carious lesions, and other abnormal oral regions. Compared with conventional image-level classification methods, object detection models can not only determine whether a lesion is present in an intraoral image but also indicate its location using bounding boxes, thereby providing more intuitive visual evidence for clinical screening and computer-aided diagnosis. Yoon et al. developed an artificial intelligence-based detection model for intraoral photographs that simultaneously localized carious regions and identified tooth numbers, demonstrating the potential of multi-object joint detection in dental image analysis (23). Xiong et al. further investigated the simultaneous detection of dental caries and pit-and-fissure sealants, enabling the model to distinguish tooth-surface regions with similar appearances but different clinical implications (24). To identify early demineralization commonly observed after orthodontic treatment, Ozsunkar et al. employed YOLOv5x to localize white spot lesions in intraoral photographs, validating the feasibility of single-stage detection frameworks for recognizing fine-grained abnormalities on tooth surfaces (25). Beyond bounding-box-based detection, Liu et al. used a deep learning-based segmentation method to simultaneously identify dental calculus, gingivitis, and dental caries, extending the capability of intraoral image models for the joint screening of multiple oral diseases (26). Zeng et al. also applied artificial intelligence to dental caries detection in intraoral photographs, further demonstrating that conventional oral photographs can serve as an important source of data for low-cost and non-invasive automated screening (27).

In recent years, research on intraoral dental image detection has gradually progressed from single-lesion localization toward fine-grained classification, multi-task analysis, and quantitative assessment. Ren et al. established a detection platform for dens evaginatus in orthodontic intraoral photographs, demonstrating that deep object detection methods can be extended to the identification of dental developmental abnormalities characterized by subtle morphological features and complex clinical manifestations (28). Yang and Chen improved the dental caries detection process through fine-grained classification and post-processing, emphasizing the importance of category-definition strategies and prediction-result optimization for reducing false-positive and false-negative detections (29). In periodontal tissue analysis, Aykol-Sahin et al. used a convolutional neural network to automatically detect and measure keratinized gingiva, extending deep learning models from lesion recognition to the quantitative assessment of oral soft tissues (30). Ramírez-Pedraza et al. employed multiple YOLO frameworks to detect dental plaque and combined the detection results with the O’Leary index to quantify oral hygiene status, demonstrating the practical value of integrating object detection results with clinical evaluation indices (31). Yin and Bao developed a YOLOv10-based dental plaque detection model for intraoral photographs of orthodontic patients, reflecting the continued development of new-generation single-stage detectors for real-time oral disease screening (32). Although existing methods have covered various targets, including dental caries, white spot lesions, dental plaque, dental calculus, and oral soft tissues, variations in illumination, saliva-induced reflections, tooth occlusion, differences in target scale, and indistinct lesion boundaries in intraoral images continue to affect detection stability. Therefore, enhancing local lesion representations, improving multi-scale feature fusion, and suppressing interference from complex backgrounds remain key challenges that require further investigation in object detection for intraoral dental images.

### 2.3 Analysis of recent dental caries detection strategies

Recent dental caries detection strategies mainly include YOLO-based single-stage detection, Transformer- or attention-based feature modeling, multi-task learning, and segmentation or fine-grained classification approaches. As summarized in Table 1, these methods have achieved promising performance in lesion recognition, localization, multi-category analysis, and computational efficiency (12, 13, 20, 22–24, 26, 29). However, their performance can still be affected by small lesion scales, indistinct boundaries, complex intraoral backgrounds, and visually similar caries appearances across different dentition types. Therefore, further improvement requires stronger modeling of dentition-level structural context together with more accurate bounding-box localization.

**Table 1:** Summary of recent strategies for dental caries detection and analysis.

Strategy	Representative studies	Performance characteristics	Remaining limitations
YOLO-based detection	(20, 22, 32)	Efficient lesion localization and favorable applicability to real-time screening	Small lesions, weak boundaries, and complex background interference remain challenging
Transformer/attention modeling	(12, 20)	Improved long-range feature interaction and discriminative representation	Fine-grained dentition structure and lesion-boundary information may still be insufficiently modeled
Multi-task detection	(23, 24)	Supports simultaneous recognition of multiple dental targets or clinically related categories	Visual similarity among targets may increase classification difficulty
Segmentation and fine-grained analysis	(13, 26, 29)	Provides more detailed lesion analysis and improves fine-grained diagnostic capability	Robustness remains sensitive to illumination, imaging conditions, and irregular lesion morphology

## 3 Method

### 3.1 Overall model architecture

This paper proposes ArchCaries-YOLO26, an end-to-end model for dental caries detection in color intraoral images. Unlike regular objects in natural scenes, carious lesions in intraoral images are generally characterized by small spatial extents, irregular morphologies, indistinct boundaries, and low contrast. Their detection is also easily affected by interdental spaces, pits and fissures, shadows, saliva-induced reflections, and soft-tissue backgrounds. Moreover, the dataset contains both caries in primary teeth and caries in permanent teeth. Although these two categories may exhibit highly similar local colors and textures, they differ considerably in crown size, spatial arrangement, adjacency relationships, and eruption status. Therefore, relying solely on local lesion features for classification and bounding-box regression can easily lead to confusion between primary and permanent tooth caries, as well as inaccurate detection boxes that are shifted, provide insufficient coverage, or include excessive normal dental tissue. To simultaneously enhance dental-arch context modeling and bounding-box localization, ArchCaries-YOLO26 adopts YOLO26 as its base framework and consists of a multi-scale feature extraction backbone, a bidirectional feature aggregation network incorporating ADT-SSM and CIBC, and an end-to-end multi-scale detection head. The overall architecture of the proposed model is illustrated in Figure 1.

**Figure 1:**
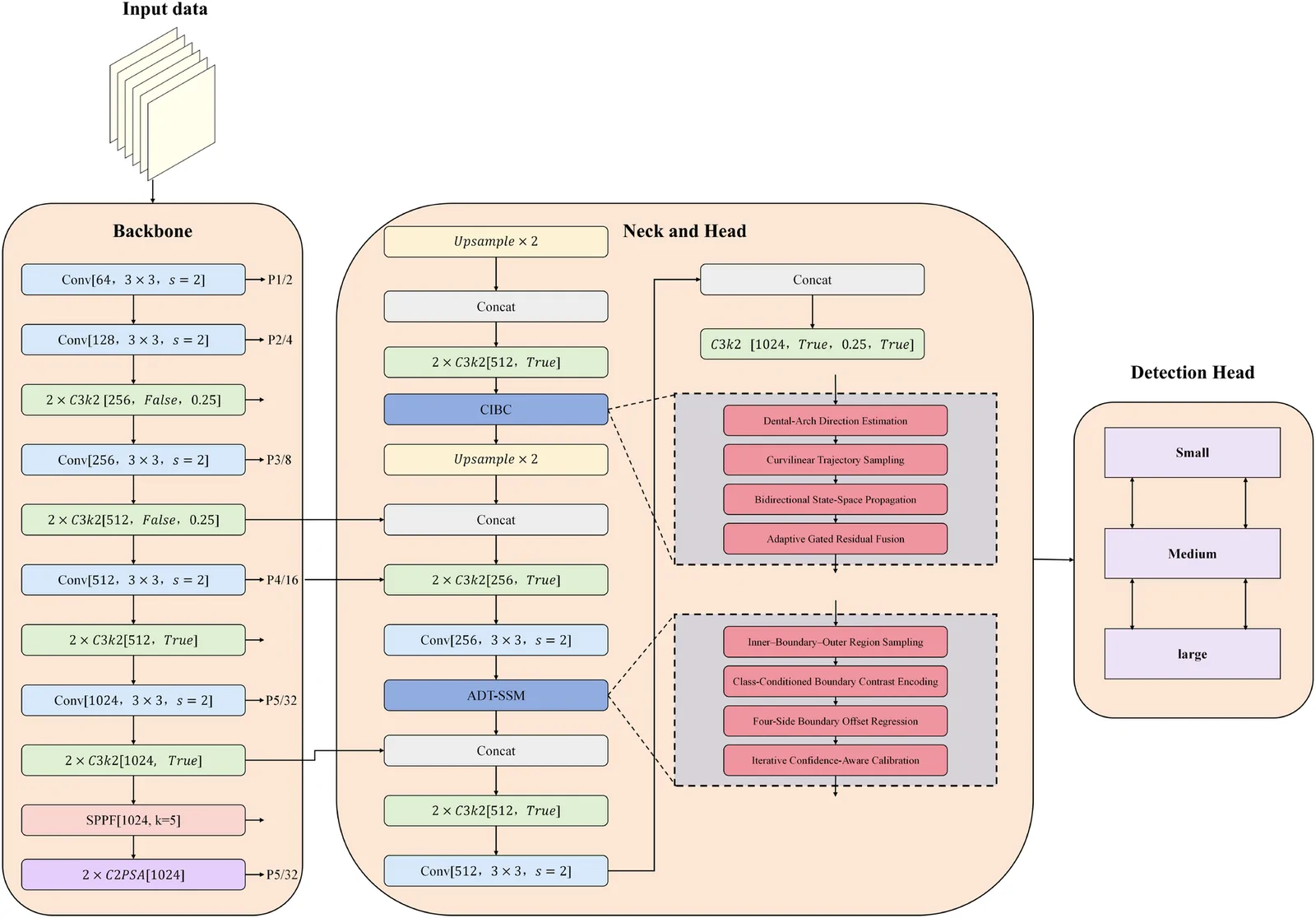
Overall architecture of the proposed ArchCaries-YOLO26, which integrates dental-arch trajectory context modeling and class-conditioned inner–outer boundary calibration into the multi-scale feature aggregation and detection framework.

The input intraoral image is first processed by consecutive convolutional downsampling operations and C3k2 feature extraction blocks, which gradually enlarge the receptive field while preserving local texture information. Subsequently, SPPF aggregates contextual information over different spatial ranges through multi-scale pooling, while C2PSA further strengthens deep semantic representations. Finally, multi-scale backbone features with strides of 8, 16, and 32 are generated. This process can be uniformly formulated as shown in Equation 1

$$F_{\mathrm{multi}}=B_{\mathrm{YOLO}26}^{\left(P3/8,\;P4/16,\;P5/32\right)}\left(I\right),$$ (1)

where I denotes the input intraoral image, $B_{\mathrm{YOLO}26}(\cdot )$ represents the backbone network composed of convolutional layers, C3k2 blocks, SPPF, and C2PSA, and $F_{\mathrm{multi}}$ denotes the multi-scale feature representation containing fine-grained spatial information, intermediate-level structural information, and high-level semantic information.

During multi-scale feature fusion, the model employs a PAN–FPN structure that combines top-down and bottom-up pathways. High-level semantic features are first upsampled and concatenated with the corresponding lateral backbone features, followed by feature reconstruction using C3k2 blocks. After the first stage of high-level semantic fusion, the Adaptive Dental-Arch Trajectory State-Space Module (ADT-SSM) is introduced. This module predicts local dental-arch orientations and curvature trends, dynamically samples potential adjacent-tooth features along the learned tooth-arrangement trajectories, and establishes continuous contextual dependencies across teeth through bidirectional state-space propagation. In this manner, structural cues such as crown size, inter-tooth distance, and dental-arch arrangement are provided to facilitate the distinction between caries in primary teeth and caries in permanent teeth. The enhanced features are then propagated toward the high-resolution feature level to preserve the fine-grained information required for detecting small carious lesions.

During the subsequent bottom-up aggregation process, the high-resolution features are mapped back to the intermediate-scale feature space through strided convolution and then fed into the Class-Conditional Inner–Outer Boundary Integral Calibration module (CIBC). This module extracts internal lesion evidence, a boundary ring region, and external dental-tissue context around each candidate region. By comparing caries-related evidence with normal tooth-surface evidence, CIBC adaptively refines the four boundaries of each candidate box according to the soft class probabilities of primary and permanent tooth caries. After multi-scale feature reconstruction, the end-to-end detection heads output class probabilities and calibrated bounding boxes at the small, medium, and large scales. The overall forward process of the model can be expressed as shown in Equation 2

$$\hat{Y}=\left(H_{S,M,L}\circ F_{\mathrm{bottom}\text{-}\mathrm{up}}^{\mathrm{CIBC}}\circ F_{\mathrm{top}\text{-}\mathrm{down}}^{\mathrm{ADT}\text{-}\mathrm{SSM}}\right)\left(F_{\mathrm{multi}}\right),$$ (2)

where $F_{\mathrm{top}\text{-}\mathrm{down}}^{\mathrm{ADT}\text{-}\mathrm{SSM}}(\cdot )$ denotes the top-down semantic propagation process incorporating ADT-SSM, $F_{\mathrm{bottom}\text{-}\mathrm{up}}^{\mathrm{CIBC}}(\cdot )$ represents the bottom-up localization enhancement process integrating CIBC, $H_{S,M,L}(\cdot )$ denotes the end-to-end detection heads designed for small-, medium-, and large-scale targets, and $\hat{Y}$ represents the final detection results for caries in primary and permanent teeth. Through this architecture, ADT-SSM primarily supplements the dental-arch context required for category discrimination by modeling tooth-arrangement relationships, whereas CIBC primarily improves the localization of irregular caries boundaries according to the differences between the internal and external regions of candidate boxes. The two modules act on semantic relationship modeling and spatial boundary calibration, respectively, thereby forming complementary feature enhancement mechanisms.

### 3.2 Class-conditioned inner–outer boundary integral calibration module, CIBC

Carious lesions in color intraoral images are typically characterized by small sizes, irregular shapes, indistinct edge transitions, and low local contrast. Their visual appearances can also be easily confused with dental pits and fissures, interproximal spaces, illumination-induced shadows, and saliva reflections. Conventional detection heads generally regress rectangular bounding-box coordinates directly from local features, without explicitly comparing lesion evidence inside the candidate box, transitional responses around its boundaries, and normal dental-tissue information outside the box. Consequently, an initial detection box may cover only the central portion of a carious lesion while omitting its peripheral regions, or it may include excessive normal dental tissue, thereby reducing localization accuracy under high intersection-over-union thresholds. Moreover, caries in primary and permanent teeth differ to some extent in crown size, tooth-surface structure, and lesion distribution. Therefore, applying an identical boundary adjustment strategy to both categories may not adequately accommodate their distinct characteristics. To address these issues, this paper proposes the Class-Conditioned Inner–Outer Boundary Integral Calibration module (CIBC). For the i-th candidate target in a multi-scale detection branch, the module receives a feature map $F\in R^{C\times H\times W}$, an initial detection box $b_{i}^{\left(0\right)}$, and an initial class-probability vector $p_{i}^{\left(0\right)}$. The initial detection box $b_{i}^{\left(0\right)}$ and class-probability vector $p_{i}^{\left(0\right)}$ correspond to the preliminary box-regression and classification outputs generated by the same multi-scale detection branch before the final prediction stage. At each detection scale, the top-16 candidates with the highest preliminary confidence scores are selected and subsequently forwarded to CIBC for calibration. It then maps the candidate-box position encoding, class semantics, and local visual features into a unified query space. The architecture of the module is illustrated in Figure 2.

**Figure 2:**
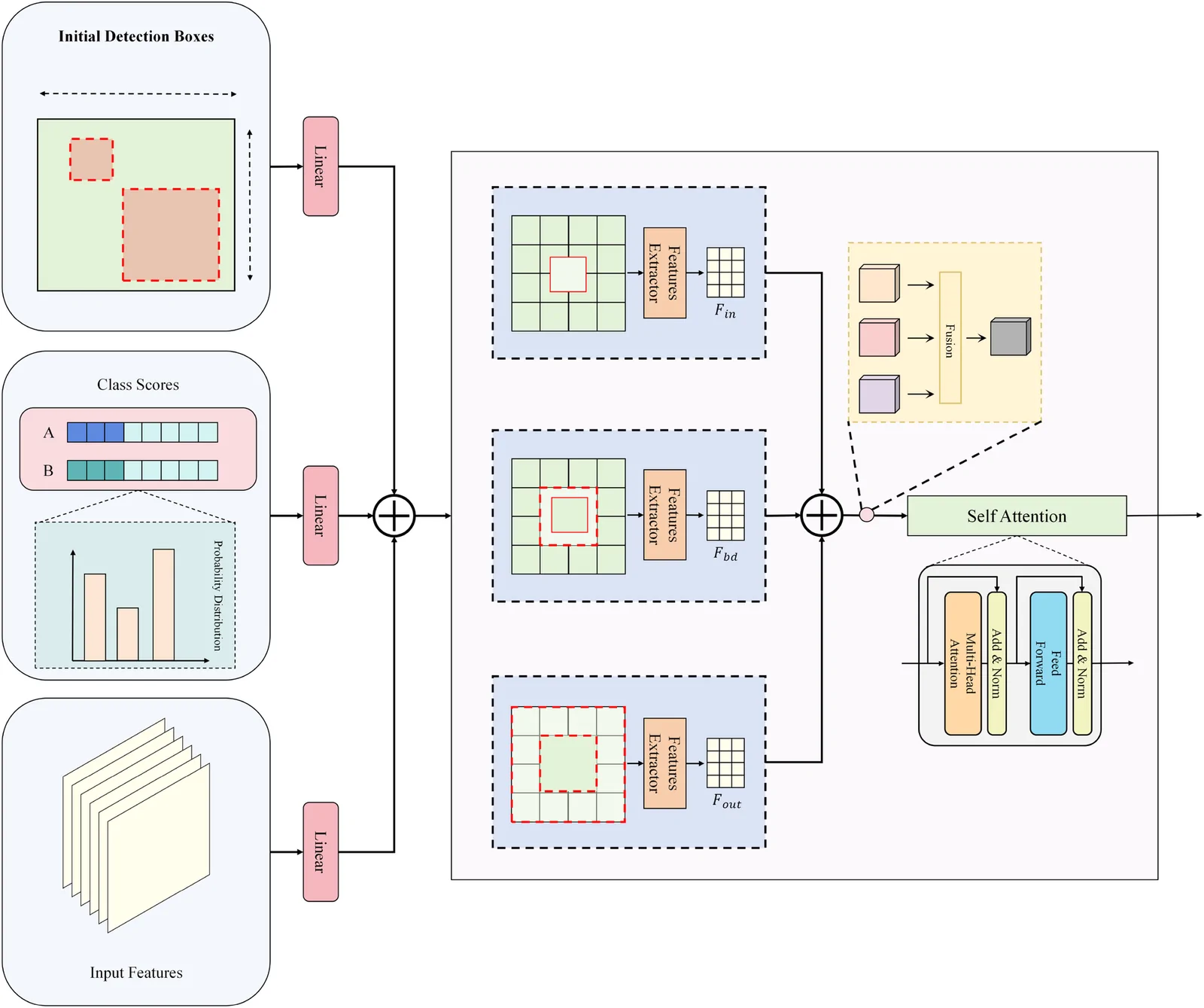
Architecture of the proposed CIBC, which jointly encodes initial detection boxes, class scores, and input features, extracts inner, boundary, and outer regional representations, and refines candidate predictions through class-conditioned feature fusion and self-attention.

The initial candidate representation is defined as shown in Equation 3

$$q_{i}^{\left(0\right)}=\phi_{\;f}\;\left(P\;\left(F,b_{i}^{\left(0\right)}\right)\right)+\phi_{b}\;\left(\mathrm{PE}\;\left(b_{i}^{\left(0\right)}\right)\right)+\phi_{c}\;\left(p_{i}^{\left(0\right)}\right),$$ (3)

where P(⋅) denotes the candidate-region feature pooling operation, PE⁡(⋅) denotes the geometric positional encoding of the detection box, and $\phi_{f}(\cdot )$, $\phi_{b}(\cdot )$, and $\phi_{c}(\cdot )$ represent the linear projections of the visual features, boundary coordinates, and class probabilities, respectively. Through this joint encoding mechanism, the subsequent calibration process can simultaneously perceive the appearance and spatial scale of the candidate region, as well as its confidence of belonging to primary-tooth caries d or permanent-tooth caries D.

After obtaining the initial candidate representation, CIBC constructs an inner lesion region, a boundary-ring region, and an outer dental-tissue region around $b_{i}^{\left(0\right)}$. The inner region is generated by proportionally shrinking the initial detection box and is used to extract core evidence with strong caries-related responses inside the candidate box. Its regional feature is expressed as shown in Equation 4

$$F_{i}^{\mathrm{in}}=P\;\left(F,R_{\mathrm{in}}\;\left(b_{i}^{\left(0\right)}\right)\right),$$ (4)

where $R_{\mathrm{in}}(\cdot )$ denotes an adaptive sampling region located inside the candidate box. The boundary-ring region covers narrow bands around the four sides of the initial box and is used to characterize the transition between carious tissue and normal tooth surfaces while preserving boundary responses in the left, top, right, and bottom directions. Its feature representation is given as shown in Equation 5

$$F_{i}^{\mathrm{bd}}=P\;\left(F,R_{\mathrm{bd}}\;\left(b_{i}^{\left(0\right)}\right)\right).$$ (5)

The outer region is formed by expanding the initial detection box outward and primarily captures non-carious information from adjacent enamel, dentin, and the local oral background. Its feature representation is defined as shown in Equation 6

$$F_{i}^{\mathrm{out}}=P\;\left(F,R_{\mathrm{out}}\;\left(b_{i}^{\left(0\right)}\right)\right).$$ (6)

Unlike methods that perform RoI feature extraction only within the candidate box, the three-layer regional sampling strategy enables the model to explicitly compare lesion responses inside the box, transitional responses around the boundary, and normal dental-tissue responses outside the box. When strong caries-related features remain outside a particular boundary, the model tends to expand the candidate box in that direction. In contrast, when a boundary region has already extended into normal dental tissue, an interdental space, or a reflective region, the model tends to contract the corresponding boundary.

To accommodate the differences in target scale and local structure between primary-tooth caries and permanent-tooth caries, CIBC uses the class probabilities to generate soft class-conditioned weights rather than selecting independent calibration branches according to discrete predicted categories. Specifically, the inner, boundary, and outer regional features are first transformed independently and are then fused using regional weights controlled by $p_{i}^{\left(0\right)}$, thereby producing a boundary representation that incorporates both class priors and inner–outer contrast information as shown in Equation 7:

$$z_{i}=q_{i}^{\left(0\right)}+\sum_{r\in \{\mathrm{in},\;\mathrm{bd},\;\mathrm{out}\}}\alpha_{i,r}\;\left(p_{i}^{\left(0\right)}\right)W_{r}F_{i}^{r},$$ (7)

where $W_{r}$ denotes the feature mapping matrix associated with region r, and $\alpha_{i,r}(\cdot )$ denotes the soft regional weight jointly generated from the probabilities of classes d and D. When the class confidence of a candidate box has not yet been fully established, the calibration information associated with the two dentition types can be continuously fused according to their predicted probabilities, thereby preventing abrupt changes in the boundary-refinement direction caused by an incorrect early-stage classification. Subsequently, the fused representations of the selected candidate boxes form the query set Z, and a self-attention encoder is employed to model the relationships among these candidate boxes. This process is expressed as shown in Equation 8

$$h_{i}=T_{\mathrm{SA}}\;\left(z_{i},Z\right),$$ (8)

where $T_{\mathrm{SA}}(\cdot )$ consists of multi-head self-attention, residual connections, normalization, and a feed-forward network. This operation allows each candidate box to use not only its own inner–outer regional differences during boundary calibration but also the scales, categories, and spatial distributions of neighboring candidate boxes, thereby reducing duplicate boxes on the same tooth and erroneous coverage across adjacent teeth.

During boundary regression, CIBC predicts offsets for the left, top, right, and bottom boundaries from the enhanced candidate representation. It further generates a four-dimensional calibration gate from the class confidence to adaptively control the magnitude of the correction in each direction. Unlike uniformly scaling the entire detection box, independent regression of the four boundaries allows the model to perform asymmetric adjustments according to the local lesion distribution. For example, when an evident caries-related response remains in the left boundary ring, the left boundary can move outward, while the right boundary, which may have already entered a normal tooth-surface region, can simultaneously contract inward. To further reduce the instability of a single boundary estimate, the module adopts an iterative confidence-calibration mechanism to progressively update the detection box. The update process at the t-th iteration is expressed as shown in Equation 9

$$b_{i}^{\left(t+1\right)}=b_{i}^{\left(t\right)}+\gamma \;\;\;\left(p_{i}^{\left(t\right)}\right)⊙W_{o}h_{i}^{\left(t\right)},\;t=0,\ldots ,T-1,$$ (9)

where $W_{o}h_{i}^{\left(t\right)}\in R^{4}$ denotes the four directional boundary offsets predicted at the t-th iteration, $\gamma (p_{i}^{\left(t\right)})\in [0,1{]}^{4}$ denotes the boundary-calibration gate jointly determined by the class information and prediction confidence, ⊙ represents element-wise multiplication, and T denotes the number of calibration iterations. After a finite number of updates, CIBC outputs the calibrated candidate boxes and forwards them to the final prediction stage. After candidate selection, regional feature extraction, class-conditioned weighting, self-attention, boundary-offset regression, confidence gating, and iterative box updates are implemented as differentiable operations, allowing gradients from the final detection objective to propagate through the calibration pathway to the corresponding feature-extraction layers. By exploiting the visual differences between the inner and outer regions of candidate boxes together with the d/D class information, CIBC improves the localization accuracy of small, irregular, and low-contrast carious lesions.

### 3.3 Adaptive dental-arch trajectory state-space module, ADT-SSM

Primary and permanent teeth in color intraoral images are not arranged along a regular horizontal or vertical grid but are continuously distributed along the distinctly curved upper and lower dental arches. During the mixed-dentition stage, the exfoliation of primary teeth, eruption of permanent teeth, and tooth replacement further lead to substantial variations in crown size, inter-tooth distance, and adjacency patterns. For small carious regions, relying solely on local color, texture, and edge information is often insufficient to determine whether a lesion is located on a primary or permanent tooth. This problem becomes more pronounced when the carious lesion is affected by shadows, pits and fissures, or saliva-induced reflections, because the local neighborhood relationships established by regular convolutions cannot adequately represent the structural continuity of teeth along the dental arch. To address this issue, this paper proposes the Adaptive Dental-Arch Trajectory State-Space Module (ADT-SSM). Let the fused feature at the current scale be denoted by $F\in R^{C\times H\times W}$. The module first employs a lightweight trajectory prediction branch composed of a 3×3 convolution, a depthwise separable convolution, and a 1×1 convolution. Without requiring additional tooth segmentation masks or tooth-position annotations, this branch generates trajectory parameters containing horizontal displacement, vertical displacement, and local curvature for each spatial position in the feature map as shown in Equation 10:

$$\Theta =\phi_{\mathrm{traj}}\;\left(F\right)\in R^{3\times H\times W},$$ (10)

where $\phi_{\mathrm{traj}}(\cdot )$ denotes the dental-arch trajectory parameter prediction network. At position $p_{0}$, Θ provides a local displacement vector $\delta_{p_{0}}$ and a curvature coefficient $\kappa_{p_{0}}$. These parameters do not directly describe the anatomical index of a specific tooth. Instead, they estimate the potential extension direction of the local tooth arrangement, allowing subsequent feature aggregation to shift from a regular two-dimensional neighborhood to adaptive propagation along the dental-arch structure.

Based on the predicted local displacement and curvature, ADT-SSM recursively generates curved sampling trajectories from each reference position $p_{0}$ along two opposite directions. As the sampling step increases, the local curvature progressively changes the displacement direction, enabling the trajectories to adapt to dental arches with different shapes in frontal, lateral, and occlusal intraoral images. The bidirectional trajectory position at the t-th step is expressed as shown in Equation 11

$$p_{t}^{\pm }=p_{0}\pm \sum_{s=1}^{t}R\;\left(s\kappa_{p_{0}}\right)\delta_{p_{0}},$$ (11)

where R(⋅) denotes a two-dimensional directional rotation transformation controlled by the local curvature, the symbols + and − correspond to the two propagation directions along the dental-arch trajectory, respectively, t∈{1,…,T}, and T denotes the trajectory sampling length. The architecture of the module is illustrated in Figure 3.

**Figure 3:**
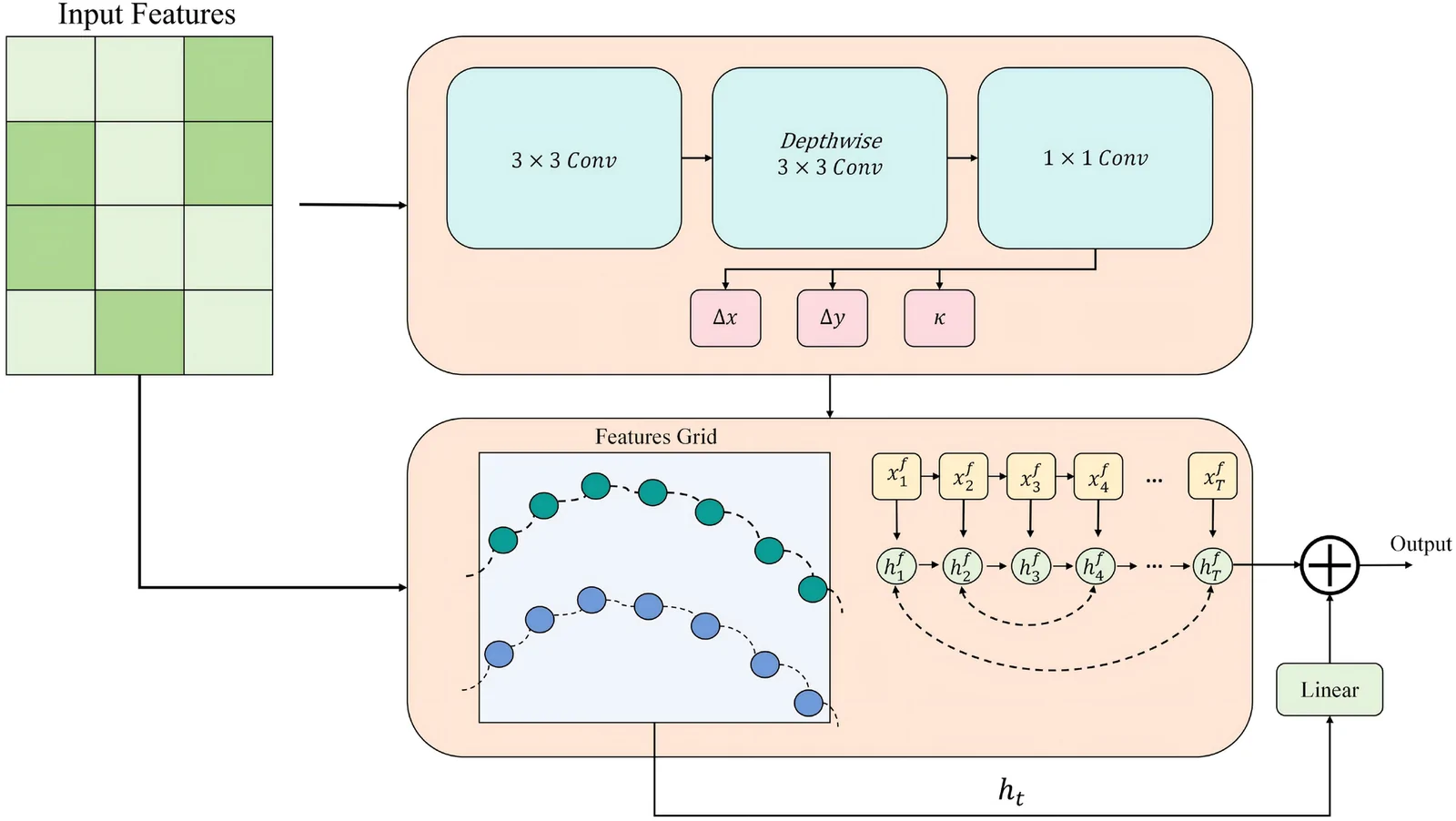
Architecture of the proposed ADT-SSM, which predicts adaptive dental-arch trajectories, samples features along curvilinear paths, and performs bidirectional state-space propagation with residual fusion to generate dentition-aware representations.

Because the generated sampling positions generally do not exactly coincide with integer feature-grid locations, the module employs differentiable bilinear interpolation to extract trajectory features from the original feature map as shown in Equation 12:

$$x_{t}^{\pm }=\sum_{q\in N(p_{t}^{\pm })}\omega \;\left(q,p_{t}^{\pm }\right)F(q),$$ (12)

where $N(p_{t}^{\pm })$ denotes the set of neighboring grid points surrounding the sampling position, and ω(⋅) denotes the bilinear interpolation weight determined by the spatial distance. The resulting trajectory sequence contains not only the caries response at the current position but also continuously aggregated information regarding the morphology, size, spacing, and arrangement variations of adjacent teeth, thereby providing structural context for distinguishing primary-tooth caries from permanent-tooth caries in mixed dentition.

After obtaining the bidirectional dental-arch trajectory sequences, the module employs selective state-space propagation instead of complete pairwise self-attention computation. Features sampled along the first direction are sequentially processed through forward state updates according to their trajectory order, allowing the current position to accumulate dental context from previously traversed locations. The propagation process is expressed as shown in Equation 13

$$h_{t}^{\;f}={\bar{A}}_{t}^{\;f}h_{t-1}^{\;f}+{\bar{B}}_{t}^{\;f}x_{t}^{+},$$ (13)

where $h_{t}^{\;f}$ denotes the forward hidden state at the t-th position, while ${\bar{A}}_{t}^{\;f}$ and ${\bar{B}}_{t}^{\;f}$ denote the state-transition and input-mapping parameters adaptively generated from the current trajectory feature. Meanwhile, features sampled along the opposite direction undergo backward state propagation to incorporate the influence of tooth arrangements on the other side of the current position as shown in Equation 14:

$$h_{t}^{b}={\bar{A}}_{t}^{b}h_{t+1}^{b}+{\bar{B}}_{t}^{b}x_{t}^{-},$$ (14)

where $h_{t}^{b}$ denotes the backward hidden state. Compared with methods that propagate information in only one direction, bidirectional state-space propagation can simultaneously capture continuous structural variations on both sides of the dental arch and avoid contextual information loss caused by differences in the scanning origin or imaging direction. Because its computational complexity increases approximately linearly with the trajectory length, this process expands the structural receptive field while preserving the lightweight characteristics of the detection network.

The forward and backward states contain complementary dentition relationships. However, different intraoral views and dentition stages may depend differently on the two propagation directions. Therefore, ADT-SSM employs an adaptive gating mechanism to dynamically fuse the bidirectional states, enabling the model to select more discriminative contextual information according to the response at the current trajectory position as shown in Equation 15:

$$z_{t}=\sigma \;\left(\phi_{\alpha }\;\left[h_{t}^{\;f}\|h_{t}^{b}\right]\right)⊙\phi_{\;f}\;\left(h_{t}^{\;f}\right)+\left[1-\sigma \;\left(\phi_{\alpha }\;\left[h_{t}^{\;f}\|h_{t}^{b}\right]\right)\right]⊙\phi_{b}\;\left(h_{t}^{b}\right),$$ (15)

where ‖ denotes channel-wise concatenation, $\phi_{\alpha }(\cdot )$ denotes the gating projection, $\phi_{f}(\cdot )$ and $\phi_{b}(\cdot )$ denote the forward- and backward-state projections, respectively, and σ(⋅) is the Sigmoid function. Subsequently, the fused trajectory sequence is remapped to the original two-dimensional space and combined with the input feature through an adaptive residual connection, producing a dentition-context-enhanced feature as shown in Equation 16:

$$F_{\mathrm{ADT}}=F+\sigma \;\left(\phi_{r}\;\left[F\|R\;\left(\{z_{t}{\}}_{t=1}^{T}\right)\right]\right)⊙\phi_{o}\;\left(R\;\left(\{z_{t}{\}}_{t=1}^{T}\right)\right),$$ (16)

where R(⋅) denotes the operation that reconstructs the trajectory sequence into a two-dimensional feature grid, $\phi_{r}(\cdot )$ denotes the residual gating projection, $\phi_{o}(\cdot )$ denotes the output projection, and $F_{\mathrm{ADT}}$ denotes the dentition-aware feature forwarded to the subsequent multi-scale fusion pathway. This residual formulation preserves the caries-related textures and local edges in the original feature while selectively injecting adjacent-tooth context propagated along the dental arch, thereby providing more stable arrangement and structural cues for the subsequent detection head to distinguish primary-tooth caries d from permanent-tooth caries D.

### 3.4 Loss function

To jointly optimize category discrimination and bounding-box localization for primary-tooth caries d and permanent-tooth caries D, this paper adopts a composite objective function consisting of a classification loss, an intersection-over-union regression loss, and a boundary-distance regression loss. During training, supervision is simultaneously applied to the one-to-one prediction branch and the one-to-many prediction branch. The one-to-one branch is consistent with the end-to-end inference process, whereas the one-to-many branch provides more sufficient positive-sample matching during the early stages of training. As training progresses, the contribution of the one-to-many branch gradually decreases, allowing the optimization emphasis to progressively shift toward the one-to-one prediction branch used for final inference. The overall training objective is expressed as shown in Equation 17

$$L_{\mathrm{total}}^{\left(t\right)}=L_{o2o}^{\left(t\right)}+\rho (t)L_{o2m}^{\left(t\right)},$$ (17)

where $L_{o2o}^{\left(t\right)}$ and $L_{o2m}^{\left(t\right)}$ denote the losses of the one-to-one and one-to-many branches at the t-th training stage, respectively, and ρ(t) denotes a branch weight that gradually decays as training proceeds. For either prediction branch r∈{o2o,o2m}, its detection objective is uniformly defined as shown in Equation 18

$$L_{r}=\lambda_{\mathrm{box}}L_{\mathrm{CIoU}}^{r}+\lambda_{\mathrm{cls}}L_{\mathrm{BCE}}^{r}+\lambda_{\mathrm{dist}}L_{L1}^{r},$$ (18)

where $\lambda_{\mathrm{box}}$, $\lambda_{\mathrm{cls}}$, and $\lambda_{\mathrm{dist}}$ control the relative contributions of the bounding-box overlap constraint, category discrimination constraint, and boundary-distance constraint, respectively.

Specifically, an adaptive class-weighted binary cross-entropy loss is employed for category prediction to supervise the confidence of each candidate position for the two caries categories d and D. Let $s_{i,c}$ denote the predicted logit of the i-th candidate position for category c, $y_{i,c}$ denote the corresponding soft matching target, and S denote the sum of all target scores. To reduce the influence of class imbalance, an adaptive class weight $\alpha_{c}^{\left(t\right)}$ is dynamically determined according to the relative frequency of each category at the current training stage, assigning a larger weight to the relatively underrepresented category. Specifically,

$$\alpha_{c}^{\left(t\right)}=\frac{{\left(n_{c}^{\left(t\right)}+\epsilon \right)}^{-1}}{\frac{1}{2}\sum_{k\in \{d,D\}}{\left(n_{k}^{\left(t\right)}+\epsilon \right)}^{-1}},\;n_{c}^{\left(t\right)}=\sum_{i=1}^{N}y_{i,c},$$ (19)

As shown in Equation 19, where $n_{c}^{\left(t\right)}$ denotes the effective number of targets belonging to category c at the current training stage, and ϵ is a small constant introduced for numerical stability. The classification loss is formulated as shown in Equation 20

$$L_{\mathrm{BCE}}=-\frac{1}{S}\sum_{i=1}^{N}\sum_{c\in \{d,D\}}\alpha_{c}^{\left(t\right)}\left[y_{i,c}\log\sigma (s_{i,c})+\left(1-y_{i,c}\right)\log\left(1-\sigma (s_{i,c})\right)\right].$$ (20)

For the positive-sample set P, the bounding-box overlap is constrained using Complete Intersection over Union, which jointly considers the overlapping area, center-point distance, and aspect-ratio difference between the predicted and ground-truth boxes as shown in Equation 21:

$$L_{\mathrm{CIoU}}=\frac{1}{S}\sum_{i\in P}w_{i}\left[1-\mathrm{CIoU}\left({\hat{b}}_{i},b_{i}\right)\right],$$ (21)

where ${\hat{b}}_{i}$ and $b_{i}$ denote the predicted and ground-truth bounding boxes, respectively, and $w_{i}$ denotes the sample weight determined by the target matching score. Meanwhile, the distances from each candidate reference point to the left, top, right, and bottom boundaries of the detection box are normalized and optimized using L1 regression, thereby directly constraining the positions of the four boundaries as shown in Equation 22:

$$L_{L1}=\frac{1}{4S}\sum_{i\in P}w_{i}{\left\|{\hat{r}}_{i}-r_{i}\right\|}_{1},$$ (22)

where ${\hat{r}}_{i}\in R^{4}$ and $r_{i}\in R^{4}$ denote the normalized predicted and ground-truth boundary distances, respectively. This distance-regression term does not model the boundary distances using discrete distributions, thereby reducing the complexity of the detection head and remaining consistent with the continuous four-boundary calibration results produced by CIBC. The CIoU term improves the overall geometric consistency between the predicted and ground-truth boxes, while the L1 distance term further stabilizes the localization of the four boundaries for small and irregular carious lesions.

## 4 Datasets and evaluation metrics

### 4.1 Datasets

#### 4.1.1 Public datasets

This study conducts experiments using the publicly available Annotated Intraoral Image Dataset for Dental Caries Detection. The dataset contains a total of 6,313 color intraoral images in JPG format, collected from participants aged 10 to 24 years in the Mithi region of Sindh Province, Pakistan. The images were captured using a Samsung Galaxy A23 mobile device and cover multiple views of the maxillary and mandibular dentitions, including occlusal, frontal, and lateral views. The dataset also includes imaging scenarios both with and without cheek retractors, thereby reflecting diverse intraoral environments under mixed-dentition and permanent-dentition conditions. The dataset was developed for the object detection of carious teeth, and dentists with clinical experience used LabelMe to annotate visible carious teeth with rectangular bounding boxes. The label d denotes caries in primary teeth, whereas the label D denotes caries in permanent teeth. The original annotations were additionally converted into YOLO, COCO, Pascal VOC, and LabelMe formats to support the training and evaluation of different object detection frameworks. In this study, the predefined training, validation, and test sets provided in the official Benchmarking Dataset are directly adopted to avoid data-distribution differences caused by a new random partition. The YOLO-format bounding boxes and category annotations are used for model training and performance evaluation. The label statistics of the dataset are shown in Figure 4.

**Figure 4:**
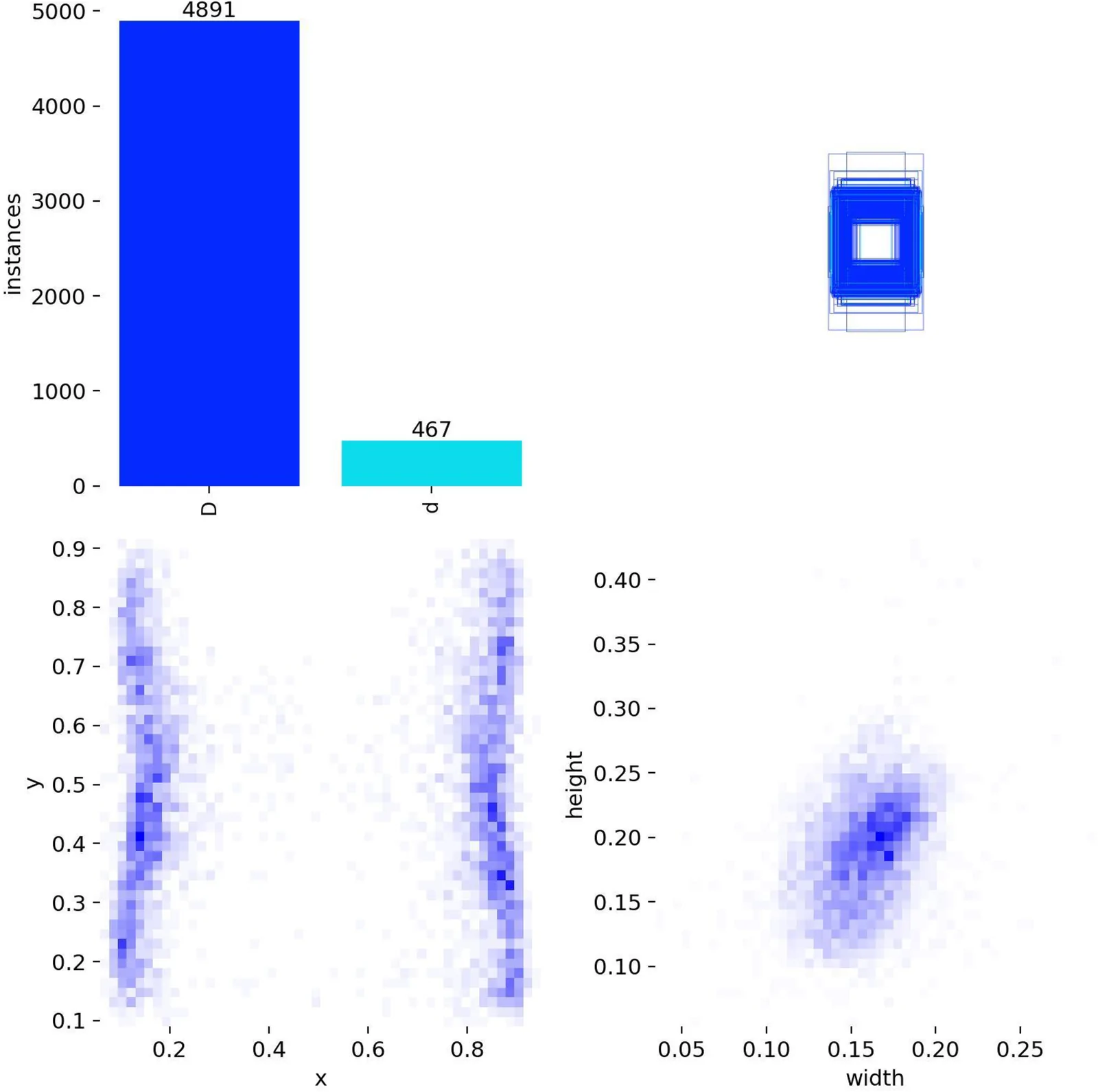
Distribution of annotated instances and normalized bounding-box characteristics in the public intraoral image dataset, including class frequencies, spatial locations, and box-size distributions. The dataset contains 4,891 permanent-tooth caries instances (D) and 467 primary-tooth caries instances (d), revealing a pronounced class imbalance, while most bounding boxes are concentrated in the bilateral tooth regions with relatively small widths and moderate heights.

#### 4.1.2 Clinical dataset

This study further constructed a clinical intraoral image dataset from Zhengzhou Central Hospital to evaluate the detection and localization performance of the proposed model for caries in primary and permanent teeth under real-world clinical conditions. The clinical dataset comprised a total of 581 color intraoral images from 137 patients, all of which were obtained during routine clinical diagnosis and treatment at Zhengzhou Central Hospital. The patients had a mean age of 11.6±4.8 years, with an age range of 3–25 years. According to dentition status, 21 patients had primary dentition, 58 had mixed dentition, and 58 had permanent dentition. The dataset was divided into training, validation, and test sets at a ratio of 7:1:2 using a patient-level partitioning strategy, ensuring that all images from the same patient were assigned exclusively to a single subset and that no patient-level overlap occurred among the three subsets. Following this patient-level partition, the training, validation, and test sets contained 403, 71, and 107 intraoral images, respectively. The training set contained 178 primary-tooth caries (d) instances and 438 permanent-tooth caries (D) instances, the validation set contained 31 d instances and 76 D instances, and the test set contained 48 d instances and 117 D instances. Overall, the clinical dataset contained 257 primary-tooth caries instances and 631 permanent-tooth caries instances, corresponding to a total of 888 annotated instances. Because this study retrospectively used clinical data generated during routine diagnosis and treatment, the reference assessment for caries annotation incorporated the corresponding clinical examination findings available at the time of diagnosis. Two dentists with 8 and 12 years of clinical experience, respectively, independently reviewed the intraoral photographs together with the available clinical examination information. The target of the object-detection task was defined as a visible carious tooth rather than the exact anatomical extent of the carious lesion. Accordingly, visible carious teeth were annotated using rectangular bounding boxes and categorized as either primary-tooth caries (d) or permanent-tooth caries (D). The bounding boxes were therefore intended to indicate the spatial locations of visible carious teeth and should not be interpreted as precise morphological or histological boundaries of carious tissue.

Inter-annotator agreement was assessed before any consensus adjudication at the tooth level. Each visible tooth was treated as an independent annotation unit, and the two dentists independently assigned its caries status and corresponding category based on their initial assessments. Cohen’s κ was calculated from these two independent sets of tooth-level categorical annotations according to $\kappa =(P_{o}-P_{e})/(1-P_{e})$, where $P_{o}$ denotes the observed proportion of agreement between the two dentists and $P_{e}$ denotes the agreement expected by chance from the marginal category distributions. The resulting Cohen’s κ was 0.69. This coefficient therefore reflects agreement in tooth-level caries/category assessment rather than the geometric overlap of the rectangular bounding boxes. Bounding-box coordinate differences were not directly included in the calculation of κ. Only after the agreement analysis had been completed were discrepant annotations, including disagreements in caries assessment, category assignment, or bounding-box placement, jointly reviewed and resolved by consensus to generate the final reference annotations used for model training and evaluation.

Because this was a retrospective study, the eligibility criteria were applied during retrieval of clinical records and intraoral images from the hospital information system rather than as post hoc exclusions after construction of the study dataset. Cases were not exported into the research dataset when the target condition could not be reliably determined from the available clinical information and intraoral photographs, including equivocal early-stage lesions with insufficient visible evidence, difficult-to-confirm interproximal lesions, or cases in which restorations, isolated tooth-surface staining, pits and fissures, or non-carious structural defects substantially prevented reliable reference assessment. Because these non-exported records were not retained as a separate research cohort, the exact numbers of excluded images, patients, and teeth could not be reliably reconstructed retrospectively. Importantly, these visual appearances were not universally removed from the included images. Pits and fissures, tooth-surface pigmentation, interdental regions, and similar non-carious structures could still be present as background or negative regions in otherwise eligible images and could therefore contribute to false-positive predictions during model evaluation. This retrospective retrieval strategy may introduce spectrum or selection bias by underrepresenting clinically equivocal, very early, or difficult-to-confirm carious presentations, and the reported performance should be interpreted in this context. Because the images were retrospectively collected during routine clinical care, detailed acquisition metadata, including the specific imaging device, camera-to-subject distance, and standardized illumination parameters, were not consistently recorded and could not be reliably recovered retrospectively. To protect patient privacy, all images and associated clinical information included in this study were strictly de-identified and anonymized prior to model training and experimental analysis, and no personally identifiable information that could directly reveal patient identity was retained. This study was reviewed and approved by the Medical Ethics Committee of Zhengzhou Central Hospital (Ethics Approval No. ZXYY2026182). All research procedures were conducted in accordance with the ethical principles of the Declaration of Helsinki, as well as applicable laws, regulations, and ethical guidelines for medical research in the People’s Republic of China. According to the determination of the ethics committee, because this study involved the retrospective use of existing clinical data that had been anonymized and de-identified, imposed no additional diagnostic or therapeutic interventions on patients, and presented minimal potential risk, the requirement for written informed consent was waived.

### 4.2 Evaluation metrics

The detection performance of all methods was evaluated using Precision, Recall, mAP@50, and mAP@50-95. These metrics are defined as shown in Equation 23:

$$\begin{array}{rl} \mathrm{Precision} & =\frac{\mathrm{TP}}{\mathrm{TP}+\mathrm{FP}},\;\mathrm{Recall}=\frac{\mathrm{TP}}{\mathrm{TP}+\mathrm{FN}},\;{\mathrm{AP}}_{c}^{\;\tau }=\int_{0}^{1}P_{c}^{\;\tau }(R)\;dR,\\ \mathrm{mAP}@50 & =\frac{1}{C}\sum_{c=1}^{C}{\mathrm{AP}}_{c}^{\;0.50},\;\mathrm{mAP}@50\text{-}95=\frac{1}{10C}\sum_{\tau \in \{0.50,0.55,\ldots ,0.95\}}\sum_{c=1}^{C}{\mathrm{AP}}_{c}^{\;\tau }, \end{array}$$ (23)

where TP, FP, and FN denote the numbers of true-positive, false-positive, and false-negative detections, respectively. $P_{c}^{\tau }(R)$ denotes the precision of category c as a function of recall R at the intersection-over-union threshold τ, and C=2 represents the two detection categories of primary-tooth caries and permanent-tooth caries.

For performance analysis, a higher Precision indicates stronger suppression of false-positive detections, whereas a higher Recall indicates a lower rate of missed carious lesions. A higher mAP@50 reflects better overall detection and classification performance under a relatively moderate localization criterion, while a higher mAP@50-95 indicates more accurate and robust bounding-box localization across increasingly stringent IoU thresholds. Therefore, these four metrics are analyzed jointly: Precision and Recall characterize the trade-off between false detections and missed lesions, whereas mAP@50 and mAP@50-95 provide complementary assessments of overall detection capability and localization accuracy, respectively.

## 5 Experimental results and analysis

### 5.1 Experimental setup and hyperparameters

To ensure a fair comparison among different methods, all experiments were conducted using the same hardware and software environment, data partition, input resolution, training length, batch size, and evaluation protocol. The detailed configurations are presented in Table 2. The architecture-specific training configurations of the comparison methods are additionally summarized in Supplementary Table A1. The experiments were performed on the Ubuntu operating system, and the models were implemented using Python and PyTorch. All models were trained and tested on a single NVIDIA A100 GPU. All input images were uniformly resized to 640×640 pixels, and the models were initialized using weights pretrained on the COCO dataset. The number of training epochs was set to 100 and the batch size was set to 32 for all methods. For the comparison methods, the publicly released architecture-specific optimizer, initial learning rate, and learning-rate schedule were retained. For ArchCaries-YOLO26 and its ablation variants, the AdamW optimizer was employed to update the network parameters. The initial learning rate and weight decay coefficient were set to $1\times 10^{-3}$ and $5\times 10^{-4}$, respectively, while the learning rate was progressively reduced using a cosine annealing schedule. Automatic mixed-precision training was enabled to reduce GPU memory consumption and improve computational efficiency. To mitigate the imbalance between primary-tooth caries (d) and permanent-tooth caries (D), a class-weighted classification loss was employed during training, assigning relatively greater importance to the minority class. In addition, data augmentation techniques, including random scaling, horizontal flipping, color perturbation, and Mosaic augmentation, were applied to improve the adaptability of the model to illumination variations, target-scale differences, and changes in imaging viewpoints in intraoral images. The model achieving the highest mAP@50-95 on the validation set was retained for final testing.

**Table 2:** Experimental environment and main training hyperparameter settings.

Configuration item	Setting
Operating system	Ubuntu 22.04 LTS
GPU	NVIDIA A100 GPU
Programming language	Python 3.10
Deep learning framework	PyTorch 2.2
Parallel computing environment	CUDA 12.1
Base detection framework	Ultralytics YOLO26M
Ultralytics version	8.4.138
Pretrained weights	COCO-pretrained weights
Input image size	640×640
ADT-SSM feature dimension	256
ADT-SSM trajectory sampling length	L=9
ADT-SSM trajectory constraints	Δx,Δy∈[−1.5,1.5], κ∈[−π/18,π/18]
CIBC feature dimension	256
CIBC attention heads	8
CIBC candidate-box generation	Preliminary multi-scale detection predictions
CIBC pooling operation	RoIAlign (7×7) + average pooling
CIBC inner-region scale	0.75× candidate-box size
CIBC boundary-region scale	1.00× candidate-box size
CIBC outer-region scale	1.25× candidate-box size
CIBC refinement iterations	2
Training epochs	100
Batch size	32
Optimizer	AdamW
Initial learning rate	$1\times 10^{-3}$
Weight decay	$5\times 10^{-4}$
Learning rate schedule	Cosine annealing
Warm-up epochs	3
Bounding-box loss weight $\lambda_{\mathrm{box}}$	7.5
Classification loss weight $\lambda_{\mathrm{cls}}$	0.5
Distance loss weight $\lambda_{\mathrm{dist}}$	1.5
Number of data-loading workers	8
Mixed-precision training	Enabled
Data augmentation	Mosaic, random scaling, horizontal flipping, and color perturbation
Inference confidence threshold	0.25
Maximum detections per image	300
Model selection criterion	Highest validation-set mAP@50-95

### 5.2 Ablation test results

To evaluate the individual contributions and collaborative effects of the Class-Conditioned Inner–Outer Boundary Integral Calibration module (CIBC) and the Adaptive Dental-Arch Trajectory State-Space Module (ADT-SSM), ablation experiments were conducted under identical data partitions, training parameters, input image sizes, and evaluation protocols. YOLO26 was adopted as the baseline model. CIBC and ADT-SSM were individually incorporated into the baseline, after which both modules were jointly introduced to construct the complete model. The experiments were performed on both the public and clinical datasets and evaluated using mAP@50, mAP@50-95, Precision, and Recall. The results are reported as the mean and standard deviation over multiple independent runs, as presented in Table 3.

**Table 3:** Ablation results of CIBC and ADT-SSM on the public and clinical datasets, reported as the mean ± standard deviation over three independent runs with different random seeds.

Method	mAP@50	mAP@50-95	Precision	Recall
Public dataset				
YOLO26	0.870±0.008	0.713±0.005	0.841±0.010	0.712±0.007
YOLO26 + CIBC	0.926±0.013	0.752±0.012	0.895±0.008	0.788±0.005
YOLO26 + ADT-SSM	0.912±0.004	0.779±0.011	0.876±0.008	0.840±0.013
Ours	0.951±0.006	0.799±0.007	0.918±0.005	0.872±0.006
Clinical dataset				
YOLO26	0.834±0.004	0.673±0.010	0.792±0.007	0.756±0.013
YOLO26 + CIBC	0.867±0.010	0.742±0.004	0.821±0.013	0.821±0.007
YOLO26 + ADT-SSM	0.899±0.007	0.718±0.008	0.771±0.013	0.883±0.011
Ours	0.920±0.006	0.776±0.007	0.779±0.009	0.905±0.009

Bold values indicate the best performance for the corresponding evaluation metric.

On the public dataset, incorporating CIBC alone improved mAP@50, mAP@50-95, Precision, and Recall by 0.056, 0.039, 0.054, and 0.076, respectively, compared with the baseline. These improvements indicate that comparing the regions inside and outside candidate boxes and calibrating their four boundaries can reduce background interference and improve caries localization. When ADT-SSM was introduced alone, Recall increased by 0.128, while mAP@50-95 increased by 0.066. This result demonstrates that propagating adjacent-tooth context along adaptive dental-arch trajectories can reduce missed detections of caries in primary and permanent teeth and improve detection performance under stricter intersection-over-union conditions. The complete model achieved the highest values for all four metrics on the public dataset, with mAP@50-95 increasing from 0.713 to 0.799.

On the clinical dataset, the complete model also achieved the highest mAP@50, mAP@50-95, and Recall, reaching 0.920, 0.776, and 0.905, respectively. However, its Precision was 0.779, which was lower than the 0.821 achieved by the model incorporating only CIBC and slightly lower than the baseline value of 0.792. This result suggests that ADT-SSM increases the model’s sensitivity to potential carious targets by enhancing dentition-context information, but it also introduces additional positive predictions under the clinical image distribution. Overall, CIBC provides more consistent improvements in localization quality and Precision, whereas ADT-SSM produces a more pronounced improvement in Recall. Their joint use achieves higher overall detection accuracy, although the balance between Precision and Recall on clinical data requires further improvement.

As shown in Table 4, the class-specific results reveal a consistent performance gap between primary-tooth caries (d) and permanent-tooth caries (D), reflecting the greater difficulty associated with the minority class. Compared with the baseline and individual-module variants, the proposed model achieves more balanced improvements in AP, Precision, Recall, and F1 for both classes across the public and clinical datasets. In particular, the improved performance for class d indicates that the combined use of CIBC and ADT-SSM helps alleviate, although does not eliminate, the adverse effect of class imbalance on minority-class detection.

**Table 4:** Class-specific results of CIBC and ADT-SSM on the public and clinical datasets.

Method	Class	AP@50	AP@50-95	Precision	Recall	F1
Public Dataset						
YOLO26	d	0.816±0.004	0.652±0.009	0.792±0.017	0.637±0.008	0.706±0.003
YOLO26	D	0.924±0.012	0.774±0.006	0.890±0.003	0.787±0.006	0.835±0.002
YOLO26 + CIBC	d	0.882±0.007	0.698±0.015	0.851±0.001	0.732±0.001	0.787±0.000
YOLO26 + CIBC	D	0.970±0.019	0.806±0.013	0.939±0.015	0.844±0.010	0.889±0.008
YOLO26 + ADT-SSM	d	0.878±0.008	0.734±0.010	0.838±0.007	0.797±0.017	0.817±0.006
YOLO26 + ADT-SSM	D	0.946±0.006	0.824±0.012	0.914±0.009	0.883±0.010	0.898±0.005
Ours	d	0.924±0.006	0.769±0.007	0.891±0.003	0.838±0.007	0.864±0.005
Ours	D	0.978±0.006	0.829±0.007	0.945±0.008	0.906±0.005	0.925±0.006
Clinical dataset						
YOLO26	d	0.794±0.004	0.622±0.011	0.746±0.014	0.721±0.011	0.733±0.005
YOLO26	D	0.874±0.004	0.724±0.015	0.838±0.005	0.791±0.015	0.814±0.007
YOLO26 + CIBC	d	0.835±0.013	0.701±0.008	0.787±0.014	0.795±0.005	0.791±0.005
YOLO26 + CIBC	D	0.899±0.007	0.783±0.005	0.855±0.012	0.847±0.011	0.851±0.005
YOLO26 + ADT-SSM	d	0.872±0.009	0.682±0.007	0.743±0.015	0.859±0.015	0.797±0.013
YOLO26 + ADT-SSM	D	0.926±0.005	0.754±0.010	0.799±0.014	0.907±0.007	0.849±0.006
Ours	d	0.896±0.007	0.748±0.003	0.748±0.005	0.884±0.012	0.810±0.004
Ours	D	0.944±0.005	0.804±0.011	0.810±0.014	0.926±0.007	0.864±0.006

Results are reported as the mean ± standard deviation over three runs.

### 5.3 Comparison of experimental results with other models

To evaluate the overall performance of the proposed model relative to existing object detection methods, this study selects several single-stage detectors, including YOLOv8, YOLOv10, YOLO11, YOLO12, and YOLO-Master, as well as Transformer-based detectors, including RT-DETR, RT-DETRV2, CPF-DETR, DEIM, RF-DETR, and STEP-DETR, for comparison. All models were trained and tested using the same data partitions, input image size, training environment, and evaluation metrics to minimize the influence of differences in experimental configurations. All results were obtained from three independent experiments conducted with different random seeds and are reported as the mean ± standard deviation. The comparative results on the public and clinical datasets are presented in Tables 5, 6, respectively.

**Table 5:** Comparative experimental results of different models on the public dataset.

Method	mAP@50	mAP@50-95	Precision	Recall
YOLOv8 (33)	0.864±0.012	0.681±0.019	0.842±0.014	0.811±0.023
YOLOv10 (34)	0.901±0.009	0.742±0.016	0.887±0.011	0.836±0.018
YOLO11 (35)	0.918±0.013	0.761±0.021	0.896±0.017	0.854±0.012
RT-DETR (36)	0.889±0.017	0.736±0.014	0.873±0.022	0.844±0.016
RT-DETRV2 (37)	0.927±0.008	0.773±0.011	0.904±0.013	0.861±0.019
YOLO12 (38)	0.912±0.015	0.782±0.018	0.891±0.009	0.865±0.014
YOLO-Master (39)	0.932±0.011	0.787±0.013	0.907±0.016	0.859±0.021
CPF-DETR (40)	0.906±0.019	0.751±0.022	0.909±0.012	0.821±0.017
DEIM (41)	0.923±0.007	0.779±0.015	0.889±0.018	0.864±0.011
RF-DETR (42)	0.936±0.014	0.788±0.020	0.913±0.015	0.856±0.013
STEP-DETR (43)	0.914±0.016	0.768±0.010	0.899±0.021	0.848±0.009
Ours	0.951±0.006	0.799±0.007	0.918±0.005	0.872±0.006

The results are reported as the mean±standard deviation over three independent runs with different random seeds.

Bold values indicate the best performance for the corresponding evaluation metric.

**Table 6:** Comparative experimental results of different models on the clinical dataset.

Method	mAP@50	mAP@50-95	Precision	Recall
YOLOv8	0.866±0.027	0.695±0.011	0.697±0.009	0.854±0.023
YOLOv10	0.801±0.022	0.631±0.024	0.679±0.025	0.738±0.026
YOLO11	0.821±0.016	0.659±0.019	0.683±0.018	0.789±0.010
RT-DETR	0.874±0.025	0.697±0.013	0.710±0.010	0.813±0.015
RT-DETRV2	0.839±0.012	0.656±0.013	0.664±0.022	0.846±0.014
YOLO12	0.886±0.015	0.720±0.012	0.707±0.016	0.848±0.022
YOLO-Master	0.807±0.018	0.649±0.022	0.685±0.025	0.780±0.023
CPF-DETR	0.847±0.027	0.704±0.007	0.712±0.023	0.867±0.019
DEIM	0.890±0.024	0.713±0.025	0.707±0.015	0.831±0.007
RF-DETR	0.817±0.018	0.637±0.024	0.699±0.019	0.808±0.011
STEP-DETR	0.872±0.016	0.733±0.026	0.687±0.017	0.815±0.024
Ours	0.920±0.006	0.776±0.007	0.779±0.009	0.905±0.009

The results are reported as the mean ± standard deviation over three independent runs with different random seeds.

Bold values indicate the best performance for the corresponding evaluation metric.

As shown in Table 5, the proposed model achieves the highest mean values for all four evaluation metrics on the public dataset. Compared with RF-DETR, which obtains the closest mAP@50 result, the proposed model improves this metric from 0.936 to 0.951, corresponding to an increase of 0.015. For the more stringent mAP@50-95 metric, the result increases from 0.788 to 0.799. Meanwhile, the proposed model achieves Precision and Recall values of 0.918 and 0.872, respectively, exceeding the corresponding best results among the comparison models by 0.005 and 0.007. Although these improvements are relatively modest, all four metrics exhibit consistent gains, and their corresponding standard deviations remain low, indicating that the model maintains relatively stable performance across the three independent runs. These results are generally consistent with the design motivations of the proposed model. ADT-SSM aggregates the arrangement and morphological information of neighboring teeth along dental-arch trajectories, providing additional contextual information for distinguishing caries in primary and permanent teeth. CIBC compares the features of the inner region, boundary ring, and outer dental-tissue region of each candidate box to calibrate the four bounding-box boundaries of irregular carious lesions. Nevertheless, the individual contributions of the two modules cannot be quantified solely from the comparisons among different models and should therefore be further evaluated in conjunction with the ablation experiments.

On the clinical dataset, the performance rankings of the different models differ to some extent from those observed on the public dataset, suggesting that differences in imaging devices, illumination conditions, acquisition angles, and oral backgrounds may affect model performance across the two datasets. As shown in Table 6, the proposed model achieves mAP@50 and mAP@50-95 values of 0.920 and 0.776, respectively, outperforming the closest comparison models by 0.030 and 0.043. Its Precision and Recall reach 0.779 and 0.905, respectively, exceeding the highest corresponding results among the other models by 0.067 and 0.038. The relatively high Recall indicates that the model can detect more potential carious targets in clinical images. However, its Precision remains considerably lower than the value of 0.918 achieved on the public dataset, suggesting that false detections caused by shadows, interdental spaces, reflections, and tooth-surface pigmentation in complex clinical environments have not been completely eliminated. Compared with detectors that rely only on feature propagation over regular grids, the dental-arch arrangement context introduced by ADT-SSM may help the model adapt to different dentition morphologies. The explicit modeling of differences between the inner and outer regions of candidate boxes by CIBC may also improve the localization quality of low-contrast carious lesions with irregular boundaries. Overall, the results on the clinical dataset demonstrate favorable detection performance under the evaluated clinical image distribution. Nevertheless, the reduction in Precision also indicates that further research is required to improve the recognition of complex interference regions and suppress false detections.

### 5.4 Qualitative experimental results

To intuitively compare the caries recognition and localization performance of different models in complex intraoral scenarios, several representative samples were selected from the public and clinical datasets for visualization. The proposed model was compared with YOLOv8, YOLOv10, RT-DETRv2, RF-DETR, STEP-DETR, and DEIM-DETR. The selected samples cover different imaging views, including maxillary occlusal, mandibular occlusal, lateral dentition, and frontal dentition views. They also contain carious targets characterized by small sizes, irregular boundaries, low local contrast, and susceptibility to interference from interdental spaces, shadows, and soft tissues. The visualization results are mainly used to examine differences among the methods in target detection, category discrimination between primary-tooth and permanent-tooth caries, and bounding-box localization. Although these qualitative results cannot replace the quantitative metrics presented above, they provide a more intuitive explanation of the performance differences among the models.

As shown in Figure 5, the predicted bounding boxes generated by the proposed model generally exhibit a high degree of consistency with the ground-truth annotations for the representative samples from the public dataset. The model also maintains relatively stable category discrimination and boundary localization across different intraoral views. Some comparison models exhibit missed detections for carious regions with small spatial extents or low local contrast. Certain models also incorrectly classify permanent-tooth caries as primary-tooth caries, indicating that the visually similar targets of classes d and D may still be confused when category prediction relies primarily on local tooth-surface textures. In addition, some predicted boxes cover relatively large regions of normal dental tissue, whereas others are positioned near the carious lesions but do not sufficiently overlap with the annotated regions. ADT-SSM aggregates the arrangement, scale, and morphological information of potentially adjacent teeth along learned dental-arch trajectories, allowing category discrimination to use contextual information beyond the individual carious region. CIBC compares the features of the inner candidate region, boundary ring, and outer tooth-surface region to perform direction-specific adjustments of the four boundaries of the initial detection box.

**Figure 5:**
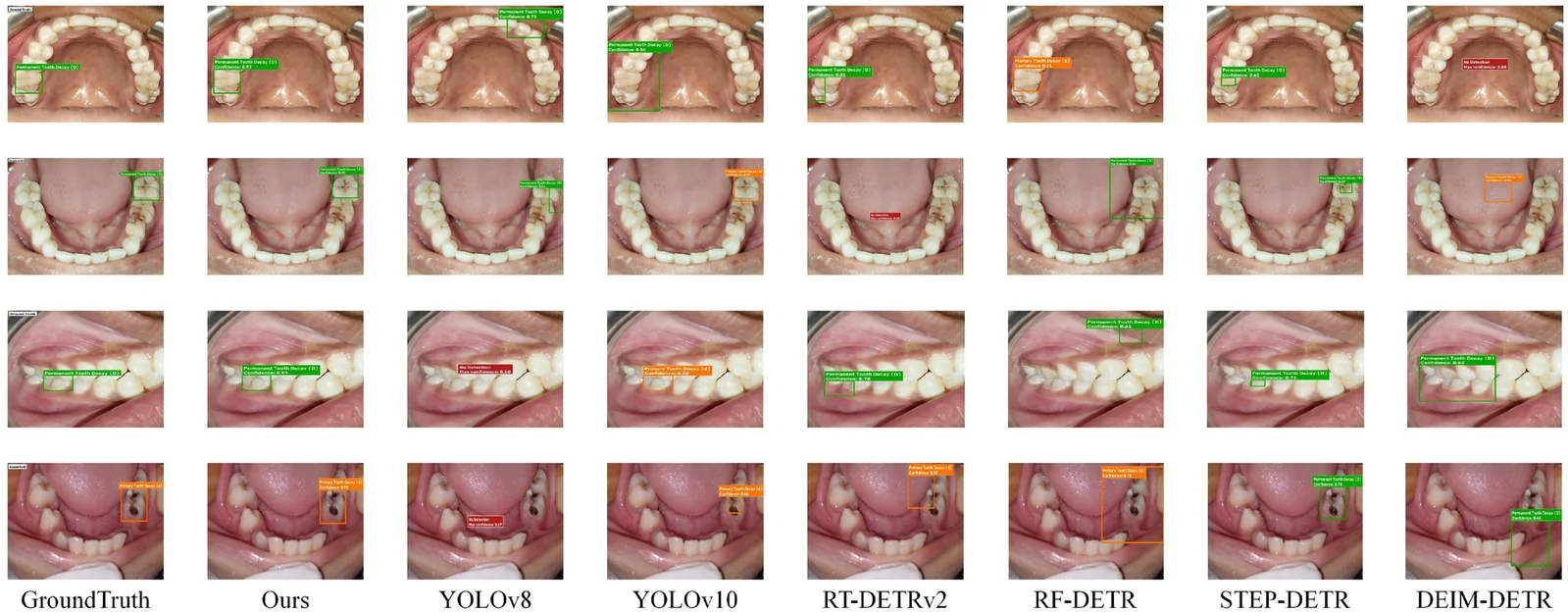
Qualitative comparison results of different object detection models on the public dataset. The green and orange bounding boxes represent predictions of permanent-tooth caries and primary-tooth caries, respectively, while the red markers indicate missed targets.

Images in the clinical dataset exhibit more pronounced variations in illumination, imaging distance, tooth arrangement, and background tissues, resulting in more complex prediction differences among the models. As shown in Figure 6, some methods fail to detect low-confidence carious lesions, small interproximal lesions, and targets located near the margins of the dentition. Other methods successfully detect the targets but exhibit incorrect category predictions, excessively large detection boxes, or substantial bounding-box displacement. For these representative samples, the proposed model successfully detects the corresponding carious regions, and its predicted bounding-box boundaries are generally close to the ground-truth boxes while maintaining relatively consistent d/D category predictions. This observation suggests that dental-arch context may help the model use information from the surrounding dentition when local textures are affected by illumination or variations in tooth-surface color. Inner–outer boundary calibration may also suppress the inclusion of irrelevant regions in the detection boxes when normal dental tissue, interdental spaces, and carious regions are spatially adjacent. Nevertheless, scale differences remain between some predicted boxes and the corresponding ground-truth boundaries, indicating that the model’s boundary estimation still has room for improvement when handling indistinct lesion margins and imaging-domain shifts in clinical images.

**Figure 6:**
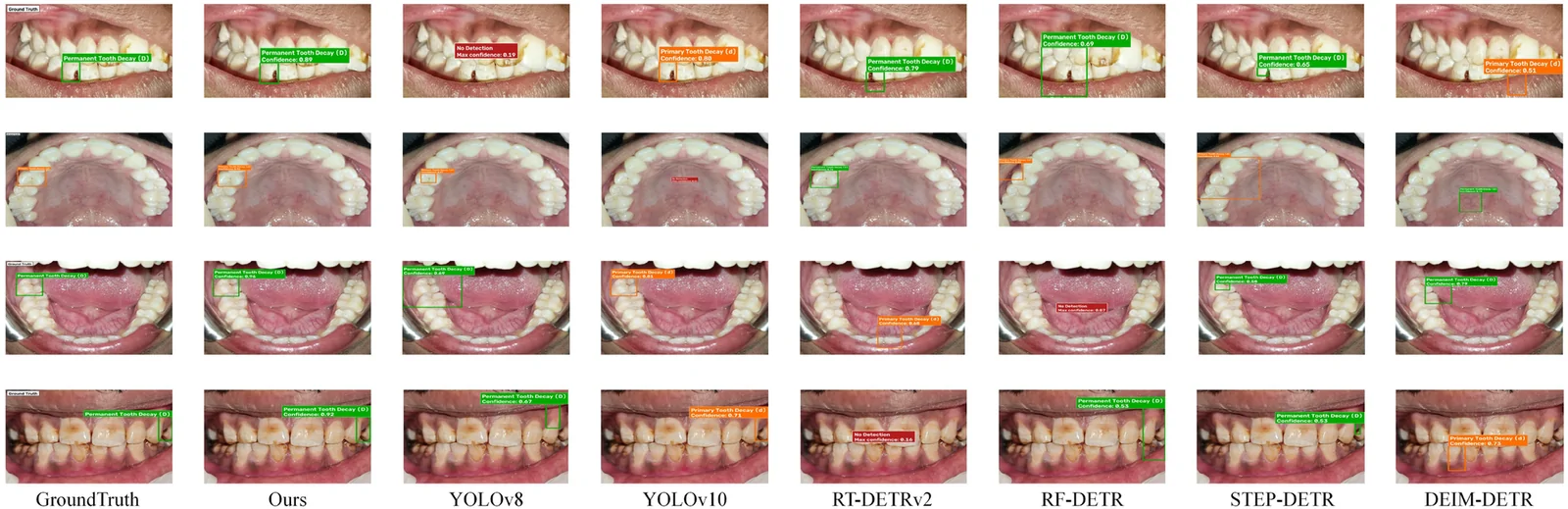
Qualitative comparison results of different object detection models on the clinical dataset, showing caries category prediction and boundary localization under different acquisition conditions and complex oral backgrounds.

### 5.5 Precision–recall curve analysis

To further analyze the detection performance of the proposed model and the baseline model for primary-tooth caries d and permanent-tooth caries D under different confidence thresholds, the precision–recall curves on the public and clinical datasets are plotted separately. The PR curve reflects the trade-off between Precision and Recall as the detection threshold changes. A curve closer to the upper-right region of the coordinate system generally indicates that the model can reduce false predictions while maintaining a high detection rate. Considering the considerable difference between the numbers of instances belonging to classes d and D in the public dataset, the class-specific curves and the overall curve for all categories are presented simultaneously to evaluate the detection stability of the model for both the majority and minority classes. The corresponding results are shown in Figure 7.

**Figure 7:**
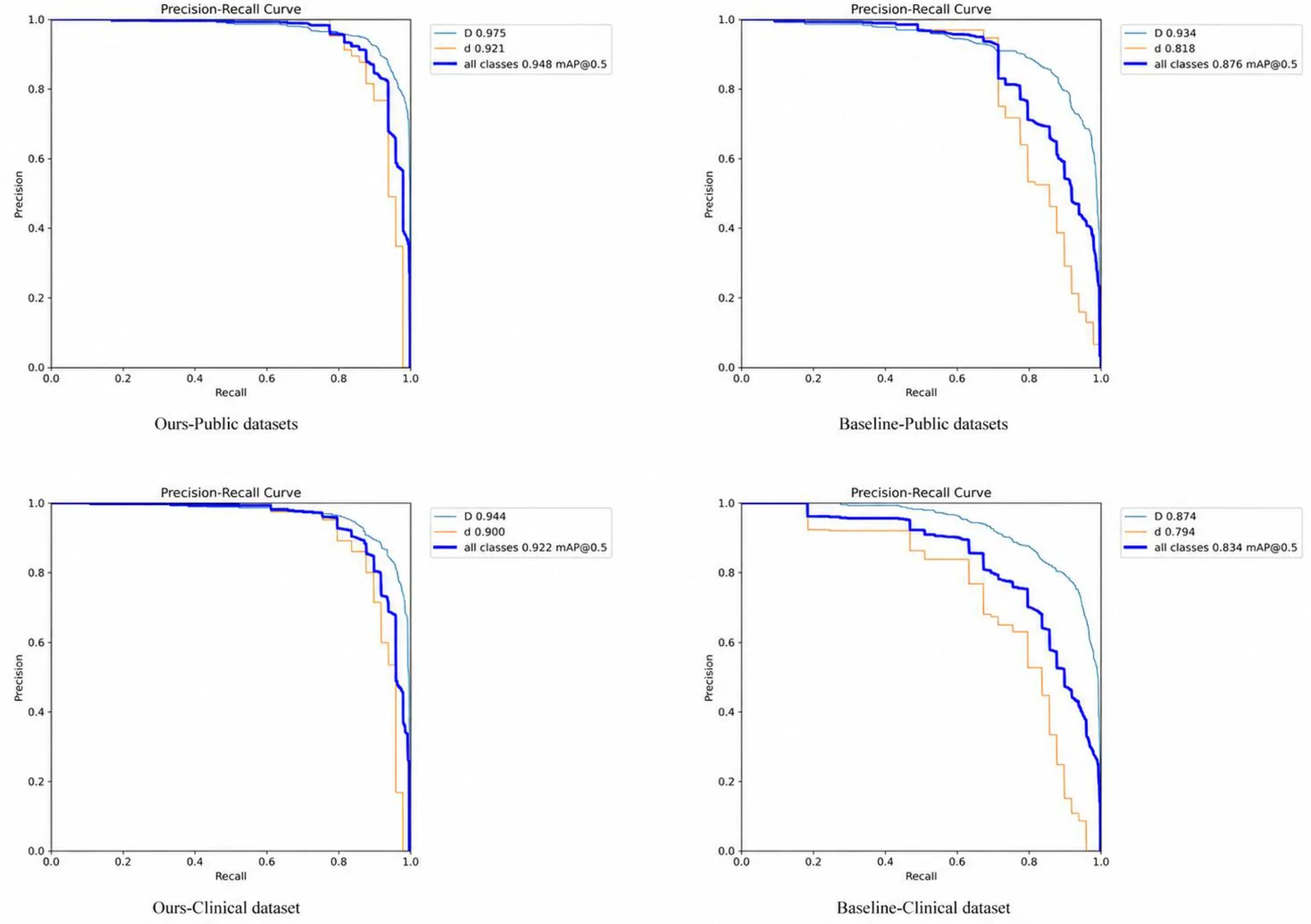
Comparison of the Precision–Recall curves of the proposed model and the baseline model on the public and clinical datasets, including the detection results for permanent-tooth caries D, primary-tooth caries d, and all categories. The curves are generated from the results of one representative run with a single random seed.

As shown in Figure 7, the overall PR curves of the proposed model on both the public and clinical datasets are generally above those of the baseline model. The proposed model also maintains relatively stable Precision as Recall increases, indicating that it can detect more carious targets while effectively controlling false detections. Compared with permanent-tooth caries D, the curve for primary-tooth caries d decreases earlier and exhibits more pronounced fluctuations. This difference may be related to the smaller number of class-d samples, larger variations in crown size, and more complex local morphology in mixed dentition. ADT-SSM propagates the arrangement and structural information of adjacent teeth along adaptive dental-arch trajectories, thereby providing additional dentition context for visually similar targets belonging to classes d and D. CIBC calibrates the bounding-box boundaries using the response differences among the inner, boundary, and outer regions of candidate boxes, which can help reduce incorrect localization caused by normal dental tissue, interdental spaces, and reflective regions. The curves on the clinical dataset exhibit more pronounced inter-class differences than those on the public dataset, indicating that complex illumination conditions, imaging angles, and changes in imaging distributions continue to affect the detection stability of the minority class. Therefore, the PR-curve results support the favorable overall classification and localization performance of the proposed model, although the detection of class d remains constrained by class imbalance and differences in clinical imaging conditions.

### 5.6 F1–confidence curve analysis

To further analyze the overall detection performance of the model under different confidence thresholds, the F1–Confidence curves of the proposed model and the baseline model on the public and clinical datasets are plotted separately. The F1 score jointly considers the balance between Precision and Recall, whereas the confidence threshold directly affects the retention and filtering of candidate targets. Therefore, the F1–Confidence curve can reflect the stability and robustness of the model under different decision thresholds, as well as performance differences between categories. Because this study involves two detection categories, namely primary-tooth caries d and permanent-tooth caries D, the F1-score trends for the two individual categories and all categories are presented simultaneously to examine their response characteristics during confidence-threshold adjustment. The corresponding results are shown in Figure 8.

**Figure 8:**
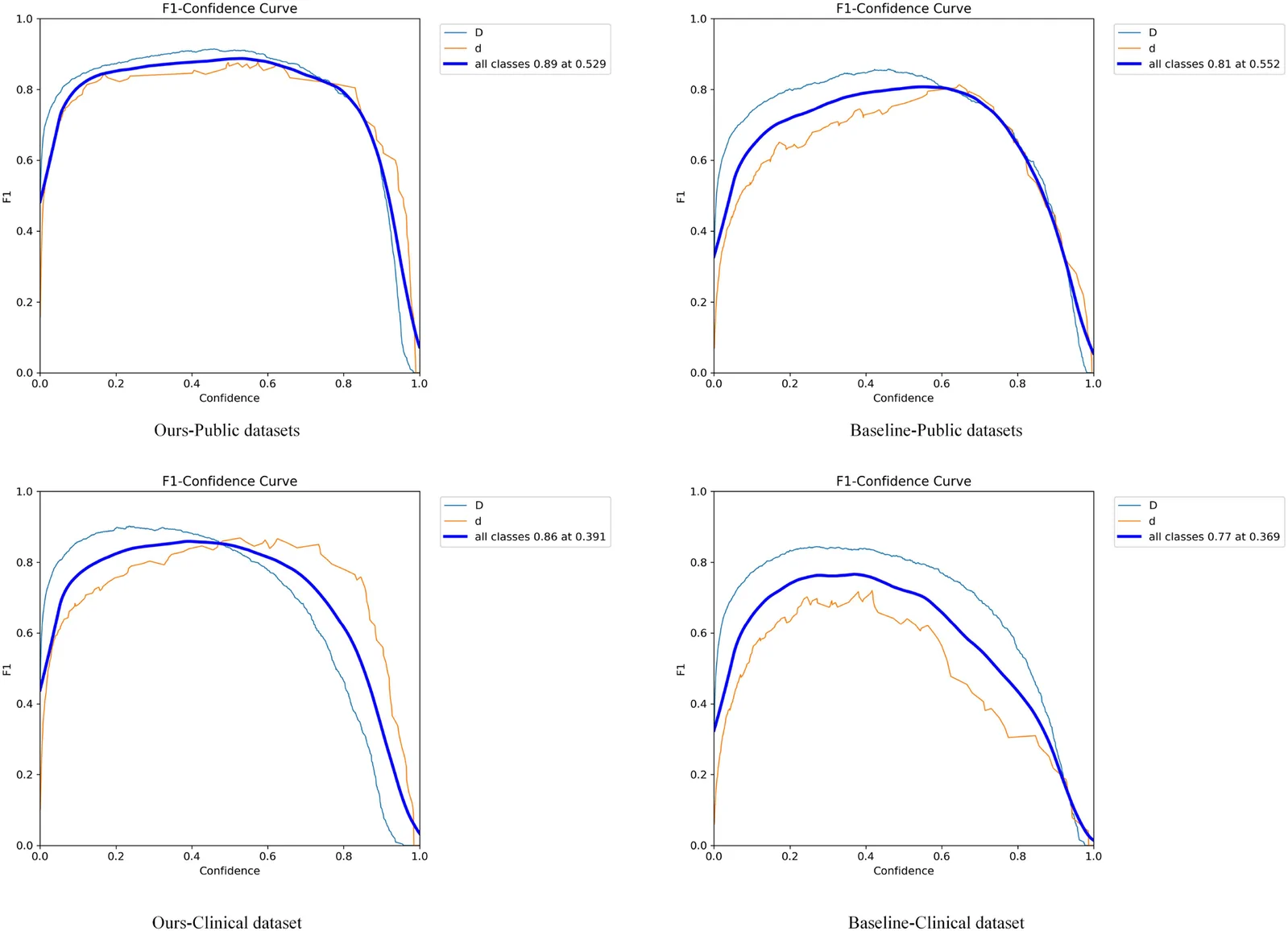
Comparison of the F1–Confidence curves of the proposed model and the baseline model on the public and clinical datasets, including the overall detection performance for permanent-tooth caries D, primary-tooth caries d, and all categories.

As shown in Figure 8, the overall F1 curves of the proposed model on the public and clinical datasets are generally higher than those of the baseline model and remain relatively stable over a broad confidence interval. This observation indicates that the proposed model can maintain a favorable balance between Precision and Recall as the threshold changes, without exhibiting substantial performance fluctuations in response to minor threshold adjustments. From a category-specific perspective, the curve for permanent-tooth caries D is generally smoother, whereas that for primary-tooth caries d exhibits relatively greater variation. This difference is consistent with factors such as the smaller number of class-d samples, more pronounced variations in target scale, and greater structural complexity in mixed dentition. With respect to the model design, ADT-SSM propagates the arrangement and morphological information of adjacent teeth along adaptive dental-arch trajectories, which helps maintain consistent category discrimination when local textures are unstable. CIBC compares the response differences among the inner, boundary, and outer regions of candidate boxes and calibrates the predicted bounding-box boundaries, thereby stabilizing the detection results under different confidence thresholds to a certain extent. Compared with those on the public dataset, the curves on the clinical dataset are more susceptible to decline and inter-class divergence, indicating that changes in imaging distributions and background interference under complex acquisition conditions continue to affect the threshold sensitivity of the minority class. Overall, the F1–Confidence curves further support the advantages of the proposed model in terms of overall detection performance and threshold stability, while also indicating that the robust detection of primary-tooth caries still has room for further improvement.

### 5.7 Sensitivity analysis of dental-arch trajectory sampling length

To analyze the effect of the dental-arch trajectory sampling length on the detection performance of ADT-SSM, a sensitivity experiment was conducted on the state-space propagation length L. The parameter L determines the range over which the module continuously samples and propagates features in two directions along the predicted dental-arch trajectory from each reference position. A smaller value of L primarily aggregates dental features near the current position, thereby preserving strong locality, but it may fail to sufficiently capture cross-tooth arrangement relationships. A larger value of L introduces broader dentition context but may also incorporate responses from distant and irrelevant teeth, soft tissues, or background regions into the propagation process. To eliminate interference from other training factors, only L was varied, while the data partition, training configuration, and evaluation protocol were kept unchanged. The results obtained with different sampling lengths on the public and clinical datasets are presented in Table 7. All results are reported as the mean ± standard deviation over three independent runs with different random seeds.

**Table 7:** Sensitivity analysis results of different dental-arch trajectory sampling lengths L on the public and clinical datasets.

L	mAP@50	mAP@50-95	Precision	Recall
Public Dataset				
5	0.925±0.008	0.766±0.007	0.894±0.006	0.851±0.008
7	0.941±0.009	0.787±0.011	0.907±0.004	0.861±0.009
11	0.936±0.007	0.777±0.010	0.898±0.010	0.866±0.004
9	0.951±0.006	0.799±0.007	0.918±0.005	0.872±0.006
Clinical Dataset				
5	0.894±0.007	0.744±0.011	0.745±0.009	0.884±0.011
7	0.911±0.009	0.761±0.009	0.759±0.008	0.877±0.004
11	0.899±0.010	0.754±0.006	0.771±0.011	0.891±0.005
9	0.920±0.006	0.776±0.007	0.779±0.009	0.905±0.009

The results are reported as the mean ± standard deviation over three independent runs with different random seeds.

Bold values indicate the best performance for the corresponding evaluation metric.

As shown in Table 7, the model maintains relatively stable detection performance under different settings of L. The standard deviations of all metrics are generally low, indicating that ADT-SSM does not exhibit pronounced random sensitivity to changes in the dental-arch trajectory sampling length. As L increases from a relatively small value, the model can capture more complete information regarding adjacent-tooth arrangements and dentition structures, resulting in improvements in most evaluation metrics. However, when the propagation length is further increased, some metrics decrease slightly, indicating that an excessively long trajectory may introduce distant dental or background features that are weakly associated with the current carious lesion, thereby weakening local discriminative information. In comparison, L=9 achieves a more balanced performance in terms of Precision, Recall, and localization accuracy on both datasets. It also exhibits relatively small variations across the three independent runs, demonstrating that this setting provides a stable balance between dental-arch context coverage and the preservation of local features. Therefore, L=9 is adopted as the default dental-arch trajectory sampling length of ADT-SSM in the subsequent experiments.

### 5.8 Grad-CAM experimental results

To further investigate the feature-attention regions and discriminative evidence employed by the proposed model during caries detection, this study adopts Gradient-weighted Class Activation Mapping (Grad-CAM) to visualize the deep feature responses of the model. Grad-CAM generates class-discriminative heatmaps by exploiting the gradient information of the target class with respect to the convolutional feature maps, thereby intuitively revealing the spatial regions on which the model primarily focuses when detecting caries in primary and permanent teeth. In this study, Grad-CAM was applied to the final feature layer of the network before the detection output, using the predicted class score of the corresponding caries category as the target for gradient computation. Considering that carious lesions in intraoral images are typically characterized by small spatial scales, irregular boundaries, and relatively low local contrast, and may also be affected by interfering factors such as interdental spaces, shadows, salivary reflections, and surrounding soft tissues, the images used for Grad-CAM visualization were randomly selected rather than manually chosen according to model performance or heatmap appearance. This analysis aims to examine the response characteristics of the model to carious lesion regions, adjacent dental structures, and surrounding contextual information, and to further investigate the detection mechanism and interpretability of the proposed model from the perspective of feature attention. The corresponding Grad-CAM visualization results are presented in Figure 9.

**Figure 9:**
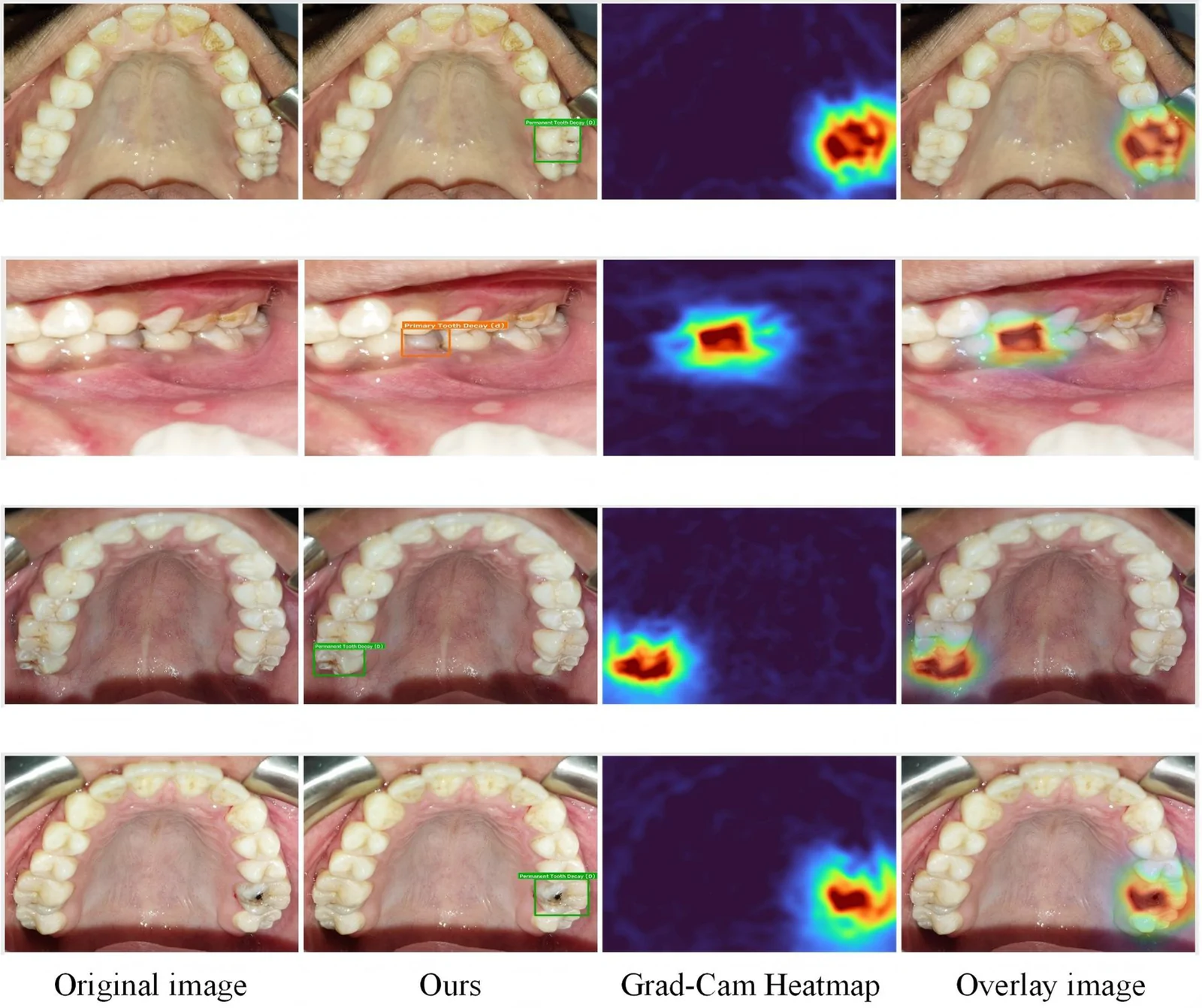
Grad-cam experimental results.

As can be observed from the Grad-CAM visualization results, across representative intraoral images with different acquisition views, dentition structures, and caries locations, the high-response regions generated by the model generally concentrate on the predicted carious lesion areas and exhibit good spatial consistency with the lesion locations identified by the detection bounding boxes. For carious lesions located in the posterior tooth region, interdental areas, and different positions along the dental arch, the major activation regions in the heatmaps are predominantly concentrated on the corresponding abnormal dental structures and their adjacent regions, whereas relatively weak responses are observed in non-target areas such as oral soft tissues, the palate, and large regions of normal tooth surfaces. This observation suggests that the model’s discrimination is not primarily dependent on irrelevant background information. Meanwhile, some high-response regions are not strictly confined to the centers of the carious lesions but extend to the surrounding dental structures and neighboring teeth to a certain extent. This pattern is consistent with the design of the proposed ADT-SSM, which models the arrangement of adjacent teeth and structural context along the dental arch, enabling the model to incorporate surrounding dentition information while focusing on local caries-related characteristics. The overlay visualizations further demonstrate a high degree of spatial overlap between the strongly activated regions and the final detection locations predicted by the model, indicating that the model is capable of concentrating visually discriminative responses on regions associated with carious lesions. From the perspective of model interpretability, these results provide intuitive visual evidence supporting the detection and localization processes of the proposed model for caries in both primary and permanent teeth.

### 5.9 Statistical and bootstrap analysis

To more comprehensively evaluate the robustness and uncertainty of the performance differences, the statistical analysis primarily focused on the comparison between ArchCaries-YOLO26 and the YOLO26 baseline. The three independent random-seed runs were retained to characterize training variability. In addition to reporting the mean and standard deviation across the three runs, the individual seed-level results and the corresponding paired differences, defined as Ours − Baseline, are explicitly reported in Table 8. In addition, RF-DETR was included in the forest-plot visualization as an additional comparison model to provide a broader view of the observed performance differences. The same paired bootstrap procedure was applied to RF-DETR, while the detailed seed-level and bootstrap results presented in Tables 8, 9 focus on the YOLO26 baseline comparison.

**Table 8:** Individual seed-level results and paired performance differences between ArchCaries-YOLO26 and the YOLO26 baseline on the public and clinical datasets.

Dataset	Metric	Baseline${}_{1}$	Ours${}_{1}$	$\Delta_{1}$	Baseline${}_{2}$	Ours${}_{2}$	$\Delta_{2}$	Baseline${}_{3}$	Ours${}_{3}$	$\Delta_{3}$	Mean Δ
Public	mAP@50	0.873	0.957	+0.084	0.876	0.948	+0.072	0.861	0.947	+0.086	+0.081
Public	mAP@50–95	0.709	0.797	+0.088	0.718	0.793	+0.075	0.711	0.807	+0.096	+0.086
Public	Precision	0.830	0.923	+0.093	0.849	0.914	+0.065	0.843	0.916	+0.073	+0.077
Public	Recall	0.718	0.869	+0.151	0.705	0.879	+0.174	0.713	0.867	+0.154	+0.160
Clinical	mAP@50	0.834	0.922	+0.088	0.830	0.913	+0.083	0.838	0.924	+0.086	+0.086
Clinical	mAP@50–95	0.668	0.774	+0.106	0.684	0.771	+0.087	0.667	0.784	+0.117	+0.103
Clinical	Precision	0.784	0.769	−0.015	0.796	0.785	−0.011	0.795	0.782	−0.013	−0.013
Clinical	Recall	0.746	0.915	+0.169	0.771	0.897	+0.126	0.750	0.903	+0.153	+0.149

**Table 9:** Paired bootstrap analysis of the performance differences between ArchCaries-YOLO26 and the YOLO26 baseline on the public and clinical datasets.

Dataset	Metric	Mean paired difference	Bootstrap 95% CI
Public	mAP@50	+0.081	[0.066, 0.095]
Public	mAP@50–95	+0.086	[0.070, 0.101]
Public	Precision	+0.077	[0.055, 0.098]
Public	Recall	+0.160	[0.137, 0.182]
Clinical	mAP@50	+0.086	[0.049, 0.119]
Clinical	mAP@50–95	+0.103	[0.065, 0.137]
Clinical	Precision	−0.013	[−0.050, 0.024]
Clinical	Recall	+0.149	[0.103, 0.190]

Because only three independent random-seed runs were available, inferential conclusions were not based solely on statistical testing across the three runs. Instead, paired bootstrap resampling of the test data was further performed to quantify the uncertainty of the observed performance differences. For the public dataset, bootstrap resampling was conducted at the image level. For the clinical dataset, resampling was conducted at the patient level so that all images belonging to the same patient remained within the same resampling unit. A total of 2,000 bootstrap replicates were generated with replacement. Within each replicate, the same resampled test units were used for the paired methods, thereby preserving the paired comparison. For each evaluation metric, the paired performance difference was calculated for each random-seed run and then averaged across the three runs. The 2.5th and 97.5th percentiles of the resulting bootstrap distribution were used to construct the corresponding 95% confidence interval. The numerical results for the comparison with the YOLO26 baseline are reported in Table 9, while the corresponding comparisons with both YOLO26 and RF-DETR are visualized in Figure 10.

**Figure 10:**
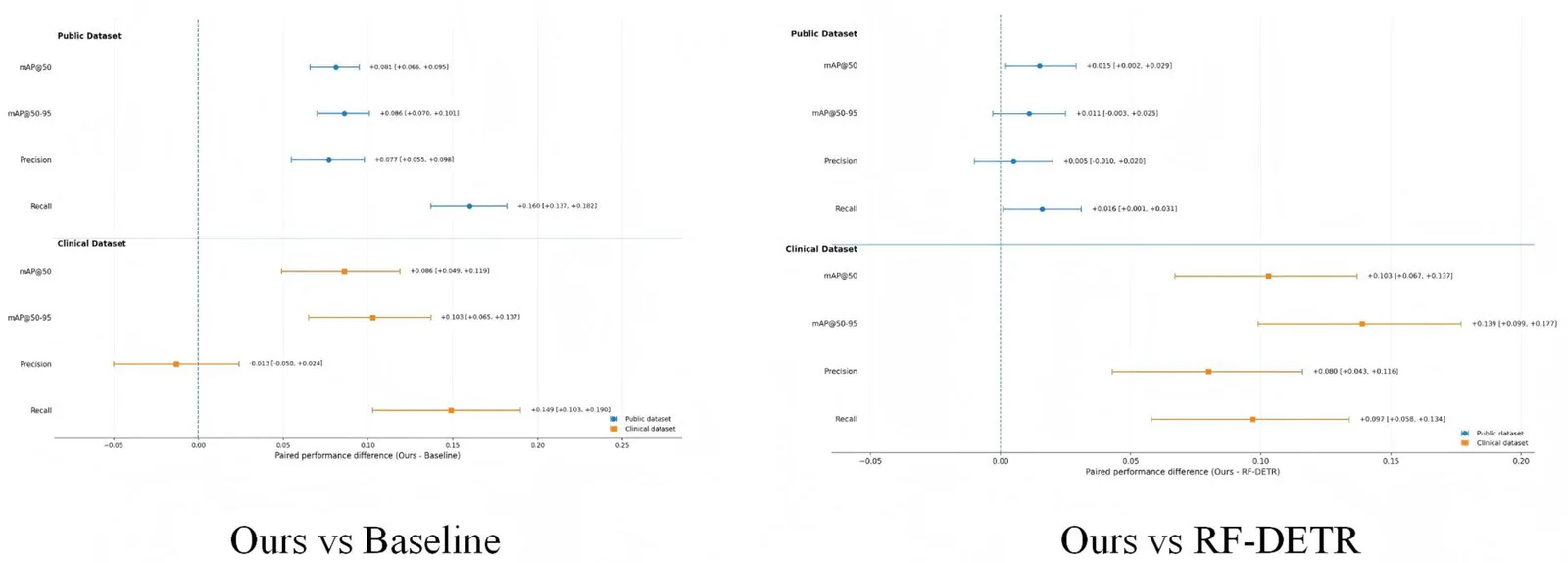
Forest plots of the paired performance differences between ArchCaries-YOLO26 and two comparison models, YOLO26 and RF-DETR, on the public and clinical datasets. Points denote the mean paired differences across three independent runs, and horizontal error bars indicate the corresponding 95% confidence intervals obtained using paired bootstrap resampling. Image-level resampling was used for the public dataset, whereas patient-level resampling was used for the clinical dataset.

As shown in Table 8, the direction of the paired differences between ArchCaries-YOLO26 and the YOLO26 baseline is generally consistent across the three independent runs. On the public dataset, the mean paired differences for mAP@50, mAP@50-95, Precision, and Recall are +0.081, +0.086, +0.077, and +0.160, respectively. The corresponding bootstrap 95% confidence intervals are [0.066,0.095], [0.070,0.101], [0.055,0.098], and [0.137,0.182], respectively. These intervals remain above zero, indicating that the direction of the observed performance gains is consistent across the resampled public test images.

On the clinical dataset, positive mean paired differences are observed for mAP@50, mAP@50-95, and Recall, with values of +0.086, +0.103, and +0.149, respectively. Their corresponding patient-level bootstrap 95% confidence intervals are [0.049,0.119], [0.065,0.137], and [0.103,0.190]. In contrast, the mean paired difference in Precision is −0.013, with a bootstrap 95% confidence interval of [−0.050,0.024], which includes zero. This result indicates that the improvement in detection sensitivity on the clinical dataset is accompanied by greater uncertainty in Precision. The additional RF-DETR comparison shown in Figure 10 provides a complementary visualization of the performance differences relative to another competitive detector under the same evaluation and bootstrap protocol.

Overall, the three independent runs demonstrate the repeatability of the observed performance trends, while the paired bootstrap analysis provides an additional characterization of uncertainty at the test-sample level. The detailed statistical results focus on the comparison with the YOLO26 baseline, while the forest plots additionally include RF-DETR to provide a broader comparison with a competitive detection model. Given the limited number of random-seed repetitions, the results are interpreted primarily according to the magnitude, direction, and bootstrap confidence intervals of the paired performance differences rather than as evidence of universal statistical superiority.

### 5.10 Error detection experiment results presentation

To provide a more comprehensive qualitative assessment of the proposed model and to avoid restricting the visualization analysis to correctly detected samples, representative error cases were additionally examined. The selected examples include false-positive, false-negative, and classification errors observed in the test set. For each case, the original intraoral image, the ground-truth annotations, and the corresponding model predictions are presented side by side. The ground-truth and predicted class labels are displayed directly above the corresponding bounding boxes, and each predicted box is additionally accompanied by its confidence score. The analysis focuses on challenging intraoral appearances in which normal anatomical structures or local visual variations may resemble carious regions, including pits and fissures, tooth-surface pigmentation, interdental spaces, illumination variations, and other low-contrast structures. The corresponding representative failure cases are presented in Figure 11.

**Figure 11:**
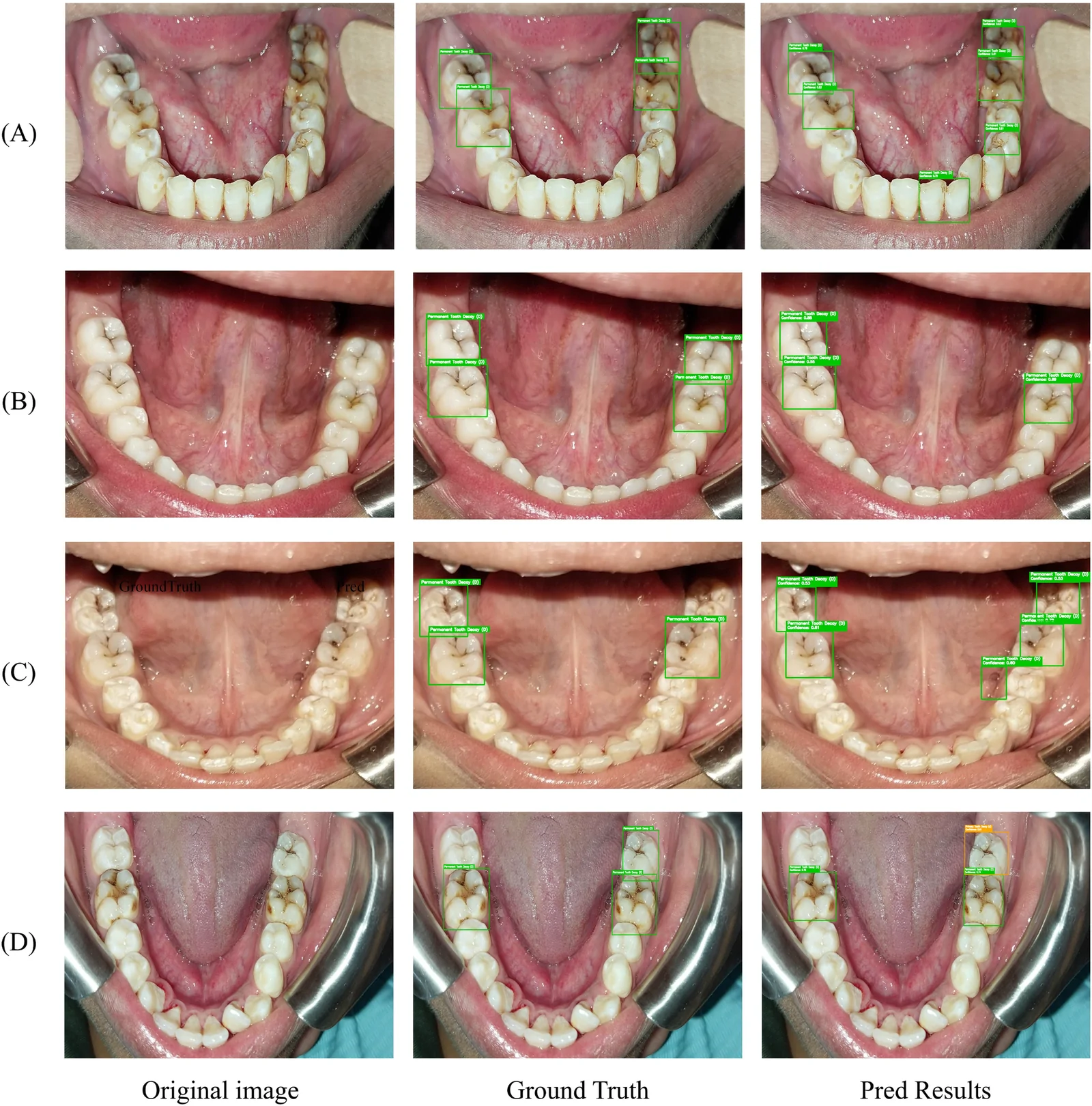
Representative error cases of ArchCaries-YOLO26 on the test set. The three columns show the original intraoral image, ground-truth annotations, and model predictions, respectively. Ground-truth boxes indicate the reference class labels, whereas each predicted box shows the predicted class together with its confidence score. **(A)** False-positive case: additional permanent-tooth caries (D) detections are produced in regions without corresponding ground-truth annotations. **(B)** False-negative case: one ground-truth permanent-tooth caries (D) target in the right posterior region is missed, while the remaining annotated targets are detected. **(C)** False-positive case: additional permanent-tooth caries (D) predictions appear in the right posterior region beyond the ground-truth annotations. **(D)** Classification-error case: a ground-truth permanent-tooth caries (D) target in the right posterior region is detected but incorrectly classified as primary-tooth caries (d), as indicated by the orange prediction box.

As shown in Figure 11A, the model correctly identifies several annotated permanent-tooth caries targets but also produces additional D predictions in regions without corresponding ground-truth boxes, representing false-positive detections. In Figure 11B, most annotated D targets are successfully detected; however, one carious tooth in the right posterior region is not detected, resulting in a false-negative case. Figure 11C presents another false-positive pattern, in which additional D predictions are generated in the right posterior dentition beyond the annotated targets. In Figure 11D, the relevant carious tooth is localized by the model, but a ground-truth D target is predicted as primary-tooth caries (d), representing a classification error. The confidence associated with each retained prediction is displayed directly in the corresponding prediction label in the figure.

These failure cases indicate that the remaining errors arise from different stages of the detection process rather than from a single source. False-positive predictions can occur when local tooth-surface appearance resembles visible carious changes, whereas false-negative predictions reflect lesions for which the available visual evidence is insufficient for reliable detection. The classification error in Figure 11D further demonstrates that visually similar appearances between primary- and permanent-tooth caries can still lead to incorrect dentition-category assignment even when the target is successfully localized. These observations indicate that the model remains sensitive to visual ambiguity in complex tooth-surface regions and that color intraoral photographs alone may not always provide sufficient information to reliably distinguish subtle carious changes from non-carious anatomical or appearance variations. The failure-case analysis therefore complements the quantitative evaluation by clarifying the specific challenging visual patterns that remain difficult for the current model to distinguish reliably.

## 6 Discussion

From the perspective of clinical application, object detection of dental caries based on color intraoral images has the advantages of relatively low acquisition cost, convenient operation, and limited dependence on specialized equipment, making it a potentially useful auxiliary tool for routine oral examinations and primary-level screening. The proposed model employs ADT-SSM to exploit the structural context of teeth arranged along the dental arch and uses CIBC to compare the regions inside and outside candidate boxes and calibrate their bounding-box boundaries. Consequently, the model can simultaneously perform category discrimination and bounding-box localization for caries in primary and permanent teeth. Experimental results on the public and clinical datasets indicate that the proposed method achieves favorable detection performance under the imaging conditions represented in the two evaluated datasets and can provide relatively intuitive information regarding the locations and categories of carious teeth. In practical applications, such models are more suitable for preliminary alerts, case prescreening, and examination assistance, such as helping clinicians rapidly locate teeth suspected of caries or providing reference information for oral health screening. It should be emphasized that model predictions cannot replace clinical diagnoses made by dentists through the combined use of visual examination, probing, and radiographic assessment. Moreover, the current within-dataset evaluations should not be interpreted as evidence of robust generalizability to unseen institutions, imaging devices, populations, or acquisition conditions.

To further examine the transferability of the proposed model across different image distributions, we conducted a cross-dataset evaluation between the public and clinical datasets. For each transfer direction, the model was trained exclusively on the training split of the source dataset, while the corresponding source validation split was used for model selection according to the highest validation mAP@50–95. The selected model was then directly evaluated on the test split of the target dataset. No information from the target dataset was used for fine-tuning, model selection, hyperparameter adjustment, or confidence-threshold tuning.

As shown in Table 10, a marked performance reduction was observed in both transfer directions compared with the corresponding within-dataset results. When trained on the public dataset and directly evaluated on the clinical dataset, the model achieved an mAP@50 of 0.724±0.019 and an mAP@50–95 of 0.547±0.023. Conversely, training on the clinical dataset and testing on the public dataset resulted in an mAP@50 of 0.681±0.024 and an mAP@50–95 of 0.506±0.018. Because no target-dataset information was involved in model optimization or selection, these reductions provide direct evidence of a domain shift between the two datasets. Differences in population characteristics, image-acquisition conditions, illumination, imaging viewpoints, and background appearance may contribute to this distributional discrepancy. Therefore, although ArchCaries-YOLO26 performs favorably within each evaluated dataset, its direct transferability across heterogeneous data sources remains limited.

**Table 10:** Cross-dataset evaluation of ArchCaries-YOLO26 between the public and clinical datasets.

Training dataset	Testing dataset	mAP@50	mAP@50–95	Precision	Recall
Public Dataset	Clinical Dataset	0.724±0.019	0.547±0.023	0.661±0.028	0.703±0.021
Clinical Dataset	Public Dataset	0.681±0.024	0.506±0.018	0.728±0.022	0.614±0.027

The model was trained using only the source-dataset training split, selected using the corresponding source validation split, and directly evaluated on the target-dataset test split without target-domain fine-tuning or tuning.

The practical value of the proposed model must still be validated through larger-scale prospective studies and real-world clinical workflows.

This study also has several objective limitations related to the available data and application environments. First, the public dataset was primarily collected from a specific region, age range, and mobile imaging device, while the clinical dataset was also relatively limited in sample size and source diversity. Therefore, the current results cannot fully represent the overall performance of the model across different populations, medical institutions, and imaging devices. Second, intraoral photographs are susceptible to variations in imaging distance, illumination intensity, saliva reflections, soft-tissue occlusion, and tooth-surface contamination. In addition, the visible characteristics of certain early-stage or interproximal carious lesions are inherently weak in color photographs, which limits the ability of methods that rely solely on visual information. Third, the number of primary-tooth caries samples is substantially smaller than that of permanent-tooth caries samples, and this class imbalance may result in greater variability in the performance estimates for the minority class. Furthermore, although the existing rectangular bounding-box annotations can indicate the approximate locations of carious teeth, they cannot fully describe the irregular morphology or actual extent of lesion invasion. Accordingly, the localization results in this study should be interpreted as bounding-box-level localization of carious teeth rather than precise delineation of the morphological boundaries of individual carious lesions. Future studies may further expand the datasets to include multiple centers, imaging devices, and age groups, introduce pixel-level segmentation masks or other fine-grained lesion annotations to characterize lesion morphology and extent more precisely, and evaluate the applicability and stability of the model in real-world screening workflows through prospective clinical trials.

## 7 Conclusion

This paper proposes ArchCaries-YOLO26 for dental caries detection in color intraoral images. The model employs ADT-SSM to model dentition context along adaptive dental-arch trajectories and uses CIBC to calibrate the four boundaries of predicted bounding boxes according to the feature differences between the inner and outer regions of candidate boxes. Experimental results on the public and clinical datasets indicate that the proposed model achieves relatively balanced performance in terms of mAP@50, mAP@50-95, Precision, and Recall. The ablation experiments further verify the complementary contributions of the two modules to category discrimination and bounding-box localization. Moreover, the qualitative results, PR curves, and F1–Confidence curves indicate relatively stable detection performance under the imaging conditions represented in the evaluated public and clinical datasets. These findings should not be interpreted as evidence of robust generalizability to unseen institutions, imaging devices, populations, or acquisition conditions. It should be noted that the localization outputs represent rectangular bounding boxes indicating the approximate locations of carious teeth and should not be interpreted as precise delineations of the irregular morphological boundaries or anatomical extent of individual carious lesions. Accordingly, ArchCaries-YOLO26 should currently be regarded as an investigational screening and decision-support tool for assisting the identification and localization of suspected carious teeth, rather than as a replacement for clinical diagnosis by dental professionals. Future work will further expand the data collected from multiple centers and imaging devices, explore pixel-level segmentation and other more fine-grained caries annotations together with tooth-position information, and validate the generalization capability and practical value of the proposed model in real-world oral health screening workflows.

## Data Availability

The original contributions presented in the study are included in the article/Supplementary Material, further inquiries can be directed to the corresponding author.
