# Supplementary material for: ArchCaries-YOLO26: a dentition-aware framework for automated detection of primary and permanent tooth caries in intraoral photographs
Source: Front Med (Lausanne). 2026 Oct 13;13:1955589. doi: 10.3389/fmed.2026.1955589 (PMC13710265; doi:10.3389/fmed.2026.1955589)
Supplement: Supplementary file 1 [file Supplementaryfile1.docx]

**Supplementary Appendix**

**ArchCaries-YOLO26: Algorithm and Experimental Configuration Details**

**A. Overall Algorithm of ArchCaries-YOLO26**

The overall procedure of ArchCaries-YOLO26 is summarized below. The framework sequentially performs multi-scale feature extraction, dental-arch context modeling, class-conditioned boundary calibration, and multi-scale caries detection.

**Algorithm A1. Overall Algorithm of ArchCaries-YOLO26**

| **Input** | Intraoral image I | |  |
| --- | --- | --- | --- |
| **Output** | Predicted caries categories and bounding boxes Ŷ | |  |
| 1 | | Resize and preprocess the input image I. | |
| 2 | | Extract multi-scale features {P3, P4, P5} using the YOLO26 backbone. | |
| 3 | | Perform top-down feature fusion through the PAN–FPN pathway. | |
| 4 | | Apply ADT-SSM to predict adaptive dental-arch trajectories. | |
| 5 | | Sample neighboring-tooth features along the predicted trajectories and perform bidirectional state-space propagation. | |
| 6 | | Fuse the propagated dentition context with the original features. | |
| 7 | | Perform bottom-up multi-scale feature aggregation. | |
| 8 | | Apply CIBC to extract inner, boundary-ring, and outer regional features for each candidate box. | |
| 9 | | Fuse the regional features using class-conditioned weights and refine the four candidate-box boundaries. | |
| 10 | | Feed the enhanced multi-scale features into the detection heads. | |
| 11 | | Generate primary-tooth and permanent-tooth caries categories and calibrated bounding boxes Ŷ. | |
| 12 | | If training: compute the classification, CIoU, and boundary-distance losses. | |
| 13 | | If training: update the model parameters by back-propagation. | |
| 14 | | Return Ŷ. | |

**B. Architecture-Specific Training Configurations**

Appendix Table A1. Architecture-specific training configurations used for the comparative experiments. All models were evaluated using the same dataset partitions and evaluation protocol, while the publicly released architecture-specific optimization settings were retained where applicable.

| **Method** | **Variant** | **Pretraining** | **Optimizer** | **Initial LR** | **Training Length** | **Batch Size** | **LR Schedule** |
| --- | --- | --- | --- | --- | --- | --- | --- |
| YOLOv8 | YOLOv8-M | COCO | AdamW | 0.01 | 100 epochs | 32 | Linear decay |
| YOLOv10 | YOLOv10-M | COCO | Adam | 0.01 | 100 epochs | 32 | Linear decay |
| YOLO11 | YOLO11-M | COCO | AdamW | 0.01 | 100 epochs | 32 | Linear decay |
| RT-DETR | RT-DETR-R50 | COCO | AdamW | 1×10⁻⁴ (1×10⁻⁵ backbone) | 100 epochs | 32 | MultiStepLR |
| RT-DETRV2 | RT-DETRv2-R50-m | COCO | Adam | 1×10⁻⁴ (1×10⁻⁵ backbone) | 100 epochs | 32 | MultiStepLR + linear warm-up |
| YOLO12 | YOLO12-M | COCO | AdamW | 0.01 | 100 epochs | 32 | Linear decay |
| YOLO-Master | YOLO-Master-EsMoE-M | COCO | Adam | 0.01 | 100 epochs | 32 | Linear decay |
| CPF-DETR | CPF-DETR | COCO | AdamW | 1×10⁻⁴ | 100 epochs | 32 | Cosine annealing |
| DEIM | DEIM-D-FINE-M | COCO | AdamW | 4×10⁻⁴ (4×10⁻⁵ backbone) | 100 epochs | 32 | Flat-cosine |
| RF-DETR | RF-DETR-M | COCO | Adam | 1×10⁻⁴ (1.5×10⁻⁴ encoder) | 100 epochs | 32 | Step |
| STEP-DETR | STEP-DETR | COCO | Adam | 1×10⁻³ | 100 epochs | 32 | No decay |
| **Ours** | **YOLO26-M + CIBC + ADT-SSM** | **COCO** | **AdamW** | **1×10⁻³** | **100 epochs** | **32** | **Cosine annealing** |

**C. Model Complexity and Computational Efficiency**

Appendix Table A2. Model complexity and computational efficiency of the compared methods. Parameter counts and FLOPs are obtained from the corresponding publicly released model configurations where available. Peak GPU memory and inference latency are measured under the same experimental environment using a single NVIDIA A100 GPU, an input size of 640×640, batch size 1, and mixed-precision inference.

| **Method** | **Variant** | **Params (M)** | **FLOPs (G)** | **Peak GPU Memory (GB)** | **Inference Latency (ms/image)** |
| --- | --- | --- | --- | --- | --- |
| YOLOv8 | YOLOv8-M | 25.9 | 78.9 | 1.74 | 4.38 |
| YOLOv10 | YOLOv10-M | 15.4 | 59.1 | 1.42 | 3.62 |
| YOLO11 | YOLO11-M | 20.1 | 68.0 | 1.56 | 3.91 |
| RT-DETR | RT-DETR-R50-m | 36.0 | 100.0 | 2.63 | 6.84 |
| RT-DETRV2 | RT-DETRv2-M | 36.0 | 100.0 | 2.51 | 6.47 |
| YOLO12 | YOLO12-M (v1.0) | 20.2 | 67.5 | 1.61 | 4.06 |
| YOLO-Master | YOLO-Master-EsMoE-M | 34.88 | 97.4 | 2.31 | 5.76 |
| CPF-DETR | CPF-DETR | 14.9 | 53.5 | 1.58 | 4.73 |
| DEIM | DEIM-D-FINE-M | 19.0 | 57.0 | 1.72 | 4.55 |
| RF-DETR | RF-DETR-M | 33.7 | 78.8 | 2.24 | 5.41 |
| STEP-DETR | STEP-DETR | 41.3 | 108.6 | 2.76 | 6.93 |
| **Ours** | **YOLO26-M + CIBC + ADT-SSM** | **23.6** | **76.4** | **1.89** | **4.72** |
